# Vascular Immune Evasion of Mesenchymal Glioblastoma Is Mediated by Interaction and Regulation of VE-Cadherin on PD-L1

**DOI:** 10.3390/cancers15174257

**Published:** 2023-08-25

**Authors:** Jing Luo, Ziyi Wang, Xuemei Zhang, Haihui Yu, Hui Chen, Kun Song, Yang Zhang, Lawrence M. Schwartz, Hongzhuan Chen, Yingbin Liu, Rong Shao

**Affiliations:** 1 Shanghai Key Laboratory of Biliary Tract Disease Research, Xinhua Hospital, Shanghai Jiao Tong University School of Medicine, Shanghai 200092, China; jluo0@bwh.harvard.edu (J.L.); yuhaihui@sjtu.edu.cn (H.Y.); chenhui2019@sjtu.edu.cn (H.C.); 2 State Key Laboratory of Oncogenes and Related Genes, Shanghai Cancer Institute, Shanghai Jiao Tong University School of Medicine, Shanghai 200025, China; wzyhaohaoxuexi@sjtu.edu.cn; 3 Department of Pharmacology and Chemical Biology, Shanghai Jiao Tong University School of Medicine, Shanghai 200025, China; yaoli@shsmu.edu.cn; 4 Department of Surgery, Brigham and Women’s Hospital, Harvard Medical School, Boston, MA 02115, USA; 5 Department of Biliary-Pancreatic Surgery, Renji Hospital, Shanghai Jiao Tong University School of Medicine, Shanghai 200127, China; 6 Department of Pathology, Shanghai General Hospital, Shanghai Jiao Tong University, Shanghai 200080, China; zhangxuemei001@126.com; 7 Nutshell Therapeutics, Shanghai 201203, China; ksong@allonutshell.com; 8 Center for Nanomedicine, Department of Anesthesiology, Brigham and Women’s Hospital, Harvard Medical School, Boston, MA 02115, USA; yzhang169@bwh.harvard.edu; 9 Department of Biology, University of Massachusetts at Amherst, Amherst, MA 01003, USA; lms@umass.edu

**Keywords:** YKL-40, VE-cadherin, vascular immune evasion, angiogenesis, PD-L1, glioblastoma (GBM)

## Abstract

**Simple Summary:**

Our study identified the primary expression of PD-L1 in vascular endothelial cells that correlated with the expression of the mesenchymal markers YKL-40 and Vim and poor patient survival. YKL-40 induced VE-cad expression, which interacted with and upregulated PD-L1, leading to vascular immune escape. In addition, upregulated VE-cad drove CCR5-mediated tumor vascularization. These data may offer solid evidence to decipher molecular mechanisms that contribute to the rigorous resistance of mesenchymal GBM to single anti-immune or angiogenic therapies. Therefore, our findings suggest a novel therapeutic strategy that combines treatments targeting both YKL-40 and VE-cad/or PD-L1 facilitates the blockade of anti-vascular immune evasion and tumor angiogenesis, benefiting patients with mesenchymal GBM.

**Abstract:**

The mesenchymal subtype of glioblastoma (mGBM), which is characterized by rigorous vasculature, resists anti-tumor immune therapy. Here, we investigated the mechanistic link between tumor vascularization and the evasion of immune surveillance. Clinical datasets with GBM transcripts showed that the expression of the mesenchymal markers YKL-40 (CHI3L1) and Vimentin is correlated with elevated expression of PD-L1 and poor disease survival. Interestingly, the expression of PD-L1 was predominantly found in vascular endothelial cells. Orthotopic transplantation of glioma cells GL261 over-expressing YKL-40 in mice showed increased angiogenesis and decreased CD8+ T cell infiltration, resulting in a reduction in mouse survival. The exposure of recombinant YKL-40 protein induced PD-L1 and VE-cadherin (VE-cad) expression in endothelial cells and drove VE-cad-mediated nuclear translocation of β-catenin/LEF, where LEF upregulated PD-L1 expression. YKL-40 stimulated the dissociation of VE-cad from PD-L1, rendering PD-L1 available to interact with PD-1 from CD8+-positive TALL-104 lymphocytes and inhibit TALL-104 cytotoxicity. YKL-40 promoted TALL-104 cell migration and adhesion to endothelial cells via CCR5-dependent chemotaxis but blocked its anti-vascular immunity. Knockdown of VE-cad or the PD-L1 gene ablated the effects of YKL-40 and reinvigorated TALL-104 cell immunity against vessels. In summary, our study demonstrates a novel vascular immune escape mechanism by which mGBM promotes tumor vascularization and malignant transformation.

## 1. Introduction

Glioblastoma (GBM) is the most lethal tumor of the central nervous system and is characterized by aberrantly elevated levels of tumor vascularization. The median survival for untreated GBM patients is about 3 months, while therapeutic intervention can increase the survival to 12–15 months [1,2]. Given the heterogeneous features of GBM, it is pathologically classified into four subtypes including classical, mesenchymal, neural, and proneural GBM [3,4]. Mesenchymal GBM (mGBM), the most common subset, expresses the mesenchymal markers Vimentin (Vim) and YKL-40 and demonstrates the most aggressive and angiogenic phenotype [5,6,7,8]. Despite employing multiple therapeutic approaches, including surgical resection, chemotherapy, radiotherapy, and targeted drugs, disease relapse occurs rapidly, and the 5-year survival rate remains at less than 3% [9,10]. Thus, there is an urgent need for alternative therapeutic strategies and/or novel therapeutic agents to improve patient outcomes.

Substantial research evidence has demonstrated that angiogenesis is essential for solid tumor development and metastasis [11,12,13,14]. Tumor vascularization is the hallmark of GBM, and a number of angiogenic factors including VEGF, bFGF, and YKL-40 have been shown to regulate vascular development in these tumors [9,15,16,17]. Elevated serum levels of YKL-40 are positively correlated with enhanced malignancy for a variety of tumors, including GBM [17,18,19]. YKL-40 functions as a pro-angiogenic factor that drives tumor angiogenesis in GBM [20,21]. In addition, YKL-40 binds to membrane receptors, including IL-13R2α and/or syndecan-1, and induces the interaction of membrane receptors with vascular endothelial cadherin (VE-cad), leading to downstream signaling activation involving β-catenin and actin in endothelial cells [22]. Therefore, the blockade of YKL-40-induced angiogenesis in the GBM xenograft has shown promising results, including reduced tumor growth, and it may offer a novel therapeutic target for clinical intervention.

Compelling evidence has recently demonstrated that anti-tumor immunity yields unprecedented rates of long-lasting tumor responses in multiple cancers [23,24,25]. Immune checkpoints such as programmed death ligand 1 (PD-L1; encoded by the CD274 gene) and PD-1 are elevated in cancers and their activated interaction results in the evasion of immune surveillance and the facilitation of tumor malignant transformation [26,27,28]. Treatment with the anti-PD-1 and PD-L1 antibody drugs ipilimumab and nivolumab has achieved favorable outcomes and has been approved as standard care for patients with a number of cancers [29,30]. However, innate or adaptive drug resistance limits the overall clinical benefit. Of note, the FDA has approved combinations of anti-angiogenic VEGF/VEGFR agents with anti-PD-1/PD-L1 antibodies for use in hepatocellular carcinoma and renal cell carcinoma [31,32]. As a result, the combination therapy has been shown to improve patients’ overall survival compared with anti-PD-L1 or PD-1 drugs alone. Mechanistically, anti-angiogenesis treatment is assumed to ablate vascularization and the interaction of adhesion molecules between vascular endothelial cells and leukocytes, thus restricting leukocyte recruitment. Nevertheless, a detailed molecular understanding of the interactions between angiogenic factors and immune checkpoints is lacking. The current study was designed to determine if YKL-40 expressed by mGBM regulates PD-L1-expressing vessels that are resistant to the anti-tumor immunity of CD8+ cytotoxic lymphocytes (CTLs). This work may help to decipher the molecular mechanisms associated with vascular immunosuppression and thus provide a novel therapeutic strategy tailored to target immune-resistant tumor vessels.

## 2. Materials and Methods

### 2.1. Cell Culture

Human microvascular endothelial cells (HMVECs) [33] were grown in EBM-2 (Lonza, Basel, Switzerland) supplemented with 5 mg/mL hydrocortisone, 10 ng/mL hEGF (Sigma-Aldrich, Burlington, MA, USA), 10% FBS, and 1% penicillin/streptomycin (ThermoFisher Scientific, Waltham, MA, USA). Human brain microvascular endothelial cells (HBMECs) (Shanghai Zeye Biotechnology), the human glioblastoma cell lines glioblastoma serum-differentiated cells (GSDCs) and YKL-40 shRNA-expressing GSDCs [34], and mouse glioma cell line GL261 cells (Shanghai Fuheng Biotechnology, Shanghai, China) were cultured in DMEM containing 10% FBS. TALL-104 cells (ATCC) were grown in RPMI 1640 medium with 10% FBS and 3.4 ng/mL IL-2 (Sigma-Aldrich). Conditioned media were collected for 24 h in FBS-free culture medium from both treated and untreated cells.

For cell co-culture systems, HMVECs and TALL-104 cells were grown in separate 60 mm plates and pre-treated with YKL-40 (200 ng/mL) for 12 h. Suspended TALL-104 cells were then transferred to the HMVEC culture for another 12 h. For BMS-1 treatment, TALL-104 cells were added to HMVEC culture in the presence or absence of a specific inhibitor of PD-L1/PD-1 interaction BMS-1 (1 μM). After 24 h, the suspended TALL-104 cells in the culture were gently washed off with PBS and collected. TALL-104 cell mRNA was isolated to evaluate the immune factor gene expression (described below).

### 2.2. Database Analyses and RNA-Sequencing

We interrogated the publicly available Gene Expression Profiling Interactive Analysis (GEPIA) dataset, which includes patient survival data (http://gepia.cancer-pku.cn, accessed on 1 January 2018). In the Single Gene Analysis mode, the expression levels of individual genes or gene pairs were determined and correlated with patient survival. We used a cutoff of *p* < 0.05.

For RNA-sequencing (RNA-seq) analysis, total RNA was isolated from HMVECs treated with or without YKL-40 (200 ng/mL) using the Trizol regent (Sangon Biotech, Shanghai, China). One microgram of total RNA per sample was sent to Beijing Genomics Institute, China for a differential transcriptome study. A total of 309 genes with a greater than two-fold change were selected for the study. GO and KEGG analyses with cellular signaling pathway enrichment were provided by the company based on the sample raw gene database. 

### 2.3. Generation of 293T Cells Stably Expressing PD-L1 and VE-Cad

cDNA for PD-L1 (full length), VE-cad (extracellular and transmembrane domains), point mutations of PD-L1 or VE-cad, and truncated PD-L1 (describe below) were individually subcloned into a retroviral pCMV-neo-vector. The viral packaging DNAs psPAX2 and pMD2.G were co-transfected with vectors containing PD-L1 or VE-cad DNAs into 293T cells using Lipo8000 (Beyotime, Shanghai, China). At 24 h after transfection, the supernatant was harvested and filtered through a filter with a 0.45 μm pore size, and the virus-containing medium in the presence of polybrene (4 μg/mL) was used to infect 293T cells. Puromycin-resistant cell populations (1 μg/mL puromycin) were used for subsequent studies.

### 2.4. Gene Knockdown

shRNA hairpins targeting human YKL-40 in GSDCs were established previously [34]. shRNA PD-L1 or VE-cad for each targeting sequence were subcloned into the pGIPZ lentiviral vector. The PD-L1 shRNA 1, 2, and 3 targeting sequences are as follows: 5′-GGGAGAATGATGGATGTGAGGACCTATATGTGGTAGAG-3′ and 5′-ACAAGCGAATTACTGTGAAGTCATCTGGACAAGCAGTG-3′ and 5′-GGACAAGCAGTGACCATCA-3′. The VE-cad shRNA 1 and 2 targeting sequences are 5′-AGCTTCACCATCAAAGTTC-3′ and 5′-CACGAAACGTGAAGTTCAA-3′. Lentiviral media were generated as described above and were used to infect HMVECs to establish the shPD-L1 or shVE-cad stable cell lines. 

### 2.5. Cross-Link Assay, Immunoblotting and Immunoprecipitation

HMVECs and TALL-104 cells were mixed (1:1) in culture for 12 h. Paraformaldehyde (4%) was added to the culture for 10 min at room temperature prior to cell lysate isolation, as described below.

For immunoblotting, cells were solubilized in ice-cold cell lysis buffer containing 6.52 mg/mL HEPES, 0.42 mg/mL NaF, 8.64 mg/mL NaCl, 0.2 mg/mL MgCl_2_, 5% NP-40, and protease inhibitor cocktail (TOPSCIENCE, Shanghai, China). Proteins were fractionated by SDS-PAGE and transferred to PVDF membrane (Millipore, Burlington, MA, USA) and then blocked in 5% milk in PBST. Subsequently, the membrane was incubated overnight at 4 °C with one of a series of primary antibodies against mouse or rabbit PD-L1 (Cell Signaling, Danvers, MA, USA), PD-1 (Cell Signaling), VE-cad (Invitrogen, Waltham, MA, USA), β-catenin (Santa Cruz Biotechnology, Santa Cruz, CA, USA), Syndecan-1 (Santa Cruz Biotechnology), LEF1 (Cell Signaling), YKL-40 (our lab), CCR5 (Abcam, Cambridge, UK), AKT, pAKT (Cell Signaling), ERK, pERK, PI3K (Santa Cruz Biotechnology), Vimentin (Dako), SMa (Abcam), E-cad (Dako, MA, USA), GAPDH (Beyotime), or β-actin (Sigma-Aldrich). The membrane was then incubated with HRP-conjugated goat anti-mouse or anti-rabbit secondary antibodies (Beyotime). The specific chemiluminescence signal was detected using the ECL reagent (ThermoFisher Scientific).

### 2.6. Cytosol and Nuclear Fragment Protein Isolation 

Confluent cells were trypsinized, and the pellet was dissolved in solution A hypotonic buffer containing 10 mM KCl, 10 mM HEPES (pH 7.8), 0.5 mM DTT, 1.5 mM MgCl_2_, 0.2 mM PMSF, and 0.5% NP-40 on ice for 15 min. The sample was subjected to centrifugation, and then supernatant was collected as the “cytosolic fraction”. The pellet was dissolved in hypertonic buffer containing 420 mM KCl, 20 mM HEPES (pH 7.8), 0.5 mM DTT, 1.5 mM MgCl_2_, 0.2 mM PMSF, 25% glycerol, and 0.2 mM EDTA on ice for 15 min. Following centrifugation, the subsequent pellet was collected as the “nuclear fraction”. 

For the co-immunoprecipitation (Co-IP) experiments, cells were lysed in immunoprecipitation lysis buffer containing 1 M Tris, 5 M NaCl, 0.84 mg/mL NaF, 0.037 mg/mL Na_3_VO_4_, 0.38 mg/mL EGTA, 0.37 mg/mL EDTA, 0.034 mg/mL PMSF, 1% Trion 100X, and 0.5% NP-40. The supernatants were incubated with the indicated antibodies for 12 h at 4 °C followed by the addition of protein G Sepharose 4 Fast Flow beads (ThermoFisher Scientific) for an additional 4 h. After washing with PBS three times, the sample was analyzed by immunoblotting, as previously described.

### 2.7. Quantitative Real-Time PCR (qPCR)

Total RNA was isolated from cells using the Trizol regent (Sangon Biotech) according to the manufacturer’s instructions. A total of 1 μg of purified total RNA was reverse-transcribed into cDNA (Accurate Biology, Changsha Hunan, China). Gene expression was assessed using the SYBROGREEN system (Accurate Biology), and the primers were as follows: human INF-γ forward 5′-GTCCAACGCAAAGCAATAC-3′ and reverse 5′-TCTTCGACCTCGAAACAG-3′; human T-bet forward 5′-TTGAGGTGAACGACGGAGAG-3′ and reverse 5′-CCAAGGAATTGACAGTTGGGT-3′; human TNF-α forward 5′-TGCTTGTTCCTCAGCCTCTT-3′ and reverse 5′-TCACCCATCCCATCTCTCT-3′; human Perforin forward 5′-GGACCAGTACAGCTTCAGCACTG-3′ and reverse 5′-AGTCAGGGTGCAGCGGG-3′; human GranzB forward 5′-GCGAATCTGACTTACGCCATTATT-3′ and reverse 5′-CAAGAGGGCCTCCAGAGTCC-3′; and human GAPDH forward 5′-TGAAGGTCGGAGTCAACGGAT-3′ and reverse 5′-CCTGGAAGATGGTGATGGGAT-3′.

### 2.8. Cell Adhesion and Migration

TALL-104 cells were added to plates of confluent HMVECs (1:1). Co-cultures were then treated with YKL-40 (200 ng/mL) and/or a CCR5 inhibitor TAK-452 (30 nM). After 8 h, the cell medium was gently aspirated, and the cells were gently washed with PBS three times. TALL-104 cells adhering to HMVECs were imaged and quantified. 

For cell migration, HMVECs (6 × 10^4^) were loaded to the top chamber of 8 µm transwell plates (Corning, Hartford, CT, USA) and incubated for 6 h at 37 °C. After washing the insert membrane, the migrated cells were fixed, stained, and quantified.

### 2.9. Cell Permeability Assay

HMVECs were cultured to confluency in the upper chamber of the 0.4 µm transwell plate and then treated with different reagents and incubated for 8 h. FITC-conjugated Dextran with 10 kDa was then added to the cell culture medium. Every 30 min, 50 µL from the bottom chamber was collected to determine the fluorescence at 485 nm/525 nm.

### 2.10. Tube Formation Assay

HMVECs (2 × 10^4^ cells) were transferred into a 96-well plate pre-coated with Matrigel (Corning, Hartford, CT, USA). After incubation for 12 h, the tube structures were visualized and analyzed with an inverted microscope (Leica, Wetzlar, Germany). The average tube number was calculated from the three fields of each sample. In order to determine the effects of TALL-104 cells on tube formation, TALL-104 cells (2 × 10^4^ cells) were mixed with HMVECs followed by loading onto the Matrigel.

### 2.11. Cell Viability Assay

Cells were grown subconfluently to determine the cell viability using the CCK-8 kit (TOPSCIENCE, Shanghai, China). HBMECs and TALL-104 cells were co-cultured, and after 12 h, TALL-104 cells were removed with PBS. The HBMEC cells attached to the plates were then used for viability assays according to the manufacturer’s instructions. 

### 2.12. Human Tissue Collection, Immunohistochemistry and Immunofluorescence

This retrospective human subject study was approved by the Ethics Committee internal review board of Shanghai General Hospital (affiliated to Shanghai Jiao Tong University). Thirty-eight patients with a primary diagnosis of GBM were enrolled, and their pathological results related to this study are presented in Table 1. The classification of mGBM depended on the expression of YKL-40 and Vim based on a semi-quantification system used to evaluate protein expression levels [20]. For the density scores, no staining is 0 points; <10% of cells stained is 1 point; 11–50% of cells stained is 2 points; and >50% of cells stained is 3 points. For the intensity: no staining is 0 points, weak staining is 1-point, moderate staining is 2 points and strong staining is 3 points. Thus, the valid range of scores was 0 to 6.

For vessel staining quantification, the VE-cad or CD31 IHC of tissue sections was quantified by the semi-quantification method described above, while CD31+ or PD-L1+ vessels were processed with immunofluorescence staining and quantified by the density of positive staining ranging from 0 to 3 points.

Paraffin-embedded tumor tissues were sectioned to 5 µm thickness and processed for immunohistochemical analysis. In brief, samples were incubated with 3% H_2_O_2_ for 30 min at room temperature to block endogenous peroxidase activity, followed by incubation with blocking buffer containing 10% goat serum for 1 h. The samples were then incubated for 2 h at room temperature with antibodies against YKL-40, Vim, hPD-L1, mPD-L1 (Cell Signaling, Danvers, MA, USA), VE-cad, hCD31 (Dako, MA, USA), mCD31, mCD34, hCD8, or mCD8 (ServiceBio, Shanghai, China). A goat anti-rabbit or mouse secondary antibody (Dako) conjugated to HRP was added for 1 h at room temperature. Finally, the DAB substrate (Dako) was introduced for several minutes, and after washing, methyl green was used for counterstaining. 

For the imaging of multiple antigens, the tissue sections were deparaffinized, and antigen retrieval was performed with the citrate antigen retrieval solution (Sangon Biotech). The samples were placed at sub-boiling temperature for 30 min followed by a cooling down period to room temperature. After incubation with blocking buffer containing 1% BSA for 1 h at room temperature, the samples were incubated overnight at 4 °C with one of the primary antibodies against hPD-L1, mPD-L1 (Cell Signaling) and hCD31 (Dako), mCD31, or mCD34 (ServiceBio). Secondary anti-rabbit Alexa Fluor 488 or anti-mouse Cy3 dye antibodies were added for 1 h at room temperature. After washing with PBS three times, the samples were incubated with DAPI solution for 10 min followed by fluorescence microscopy.

### 2.13. Immunocytochemistry

Cells were grown to confluency and fixed with 4% paraformaldehyde for 10 min at room temperature, blocked with 1% BSA, and then incubated with one of the primary antibodies including LEF1, PD-L1, β-catenin, VE-cad, Vim or Ki67 (ThermoFisher Scientific) at 4 °C overnight. After washing with PBS, the cells were incubated with an Alexa 488 or a Cy3 secondary antibody at room temperature for 1 h. Images were detected by a confocal laser scanning microscope (Leica). 

### 2.14. Dual Luciferase Reporter Assay

Human CD274 promoter DNA (+1 to −600 bp) with wild type (ACAAAG) or mutant (ACAGGA) at the −483 bp TCF/LEF binding motif was cloned into the pGL3 basic vector. The 293T cells were co-transfected with 2 µg of the pGL3 empty vector, pGL3 WT TCF/LEF, or pGL3 TCF/LEF mutant DNA in the presence of Lipo8000 (Beyotime). After 24 h of incubation, luciferase activity was measured using the dual luciferase reporter assay according to the manufacturer’s instructions (Promega, Madison, WI, USA).

### 2.15. Intracranial Xenograft Tumor Models 

All animal experiments were approved by the Institutional Animal Care and Use Committee (IACUC) of Shanghai Jiao Tong University School of Medicine. For GL261 cell transplantation, we utilized 4-week-old C57BL/6 mice that contained an intact immune system. GL261 cells expressing either the empty vector or YKL-40 (3 × 10^5^ cells in 10 μL PBS) (n = 6/group) were injected into mouse brains at a controlled flow rate of 2 μL/min by a stereotactic frame (RWD Life Science, San Diego, CA, USA). Mice displayed labored breath and difficulty moving between weeks 3 and 4 after transplantation, at which point they were sacrificed, and brain tumors were collected.

### 2.16. Statistical Analysis

GraphPad Prism 8 software was used to perform the statistical analysis and to generate graphs/and plots. Error bars represent the standard error of the mean (SEM). Statistical significance was determined by two-tailed unpaired Student’s t test. Non-parametric tests were used for non-log-transformed gene expression data from the TCGA database. Statistical significance is indicated in the figures, as follows: 0.01 ≤ * *p* < 0.05; 0.001 ≤ ** *p* < 0.01; *** *p* < 0.001; **** *p* < 0.0001.

## 3. Results

### 3.1. YKL-40 Expressed by Tumor Cells Is Correlated with Vascular PD-L1 Levels in mGBM

**Figure 1:**
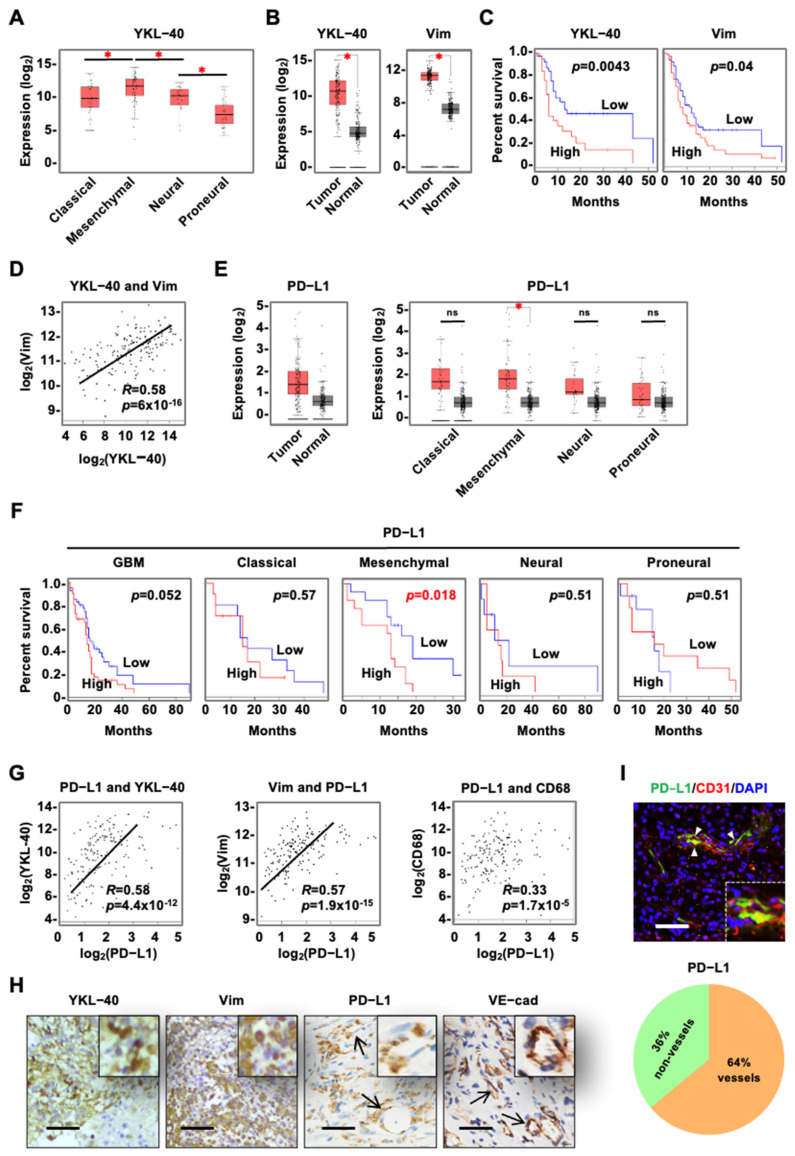
YKL-40 and Vimentin expression levels are correlated with PD-L1 expression, which is largely localized to vascular endothelial cells in mGBM. (**A**) Database analysis (http://gepia.cancer-pku.cn, accessed on 1 January 2018) of YKL-40 levels in four subtypes of GBM, including classical (n = 40), mesenchymal (n = 55), neural (n = 28), and proneural (n = 37) GBM, * *p* < 0.05. (**B**) Analysis of YKL-40 and Vim levels in GBM (normal = 207, tumor = 163), * *p* < 0.05. (**C**) High levels of YKL-40 and Vim were correlated with reduced disease-free survival in patients with GBM (YKL-40, n = 41; VIM, n = 81). (**D**) Correlation between YKL-40 and Vim in GBM samples (n = 163). (**E**) Analysis of the PD-L1 level in GBM (normal = 207, tumor = 163) and four subtypes of GBM, * *p* < 0.05. (**F**) Relationship between the PD-L1 level and the poor prognosis of general (n = 40), classical (n = 10), mesenchymal (n = 14), neural (n = 7) and proneural (n = 9) GBM cases. (**G**) Correlations between PD-L1 and YKL-40, Vim, or CD68 in GBM (n = 163). (**H**) IHC analyses of YKL-40, Vim, PD-L1, and VE-cad levels in mGBM samples (n = 26). The arrowheads indicate PD-L1+ and VE-cad+ vessels. Inserts showed large positive signals. Scale bar, 50 μm. (**I**) Dual immunofluorescence staining of PD-L1 (green) and CD31 (red) in mGBM samples that expressed PD-L1 (score ≥ 3 in Table 1) (n = 6) from a total of 10 cases of PD-L1-positive GBM. An insert showed overlapping vessels. PD-L1-positive vessels (0–3 points) indicated by arrow heads were quantified from the vessels co-expressing CD31 and PD-L1 in a total of PD-L1-positive cells that included vascular and non-vascular cells. Scale bar, 50 μm.

Glioblastoma (GBM) has been classified into four pathological subtypes including classic, mesenchymal, neural, and proneural. The mGBM subtype is the most aggressive and angiogenic and is characterized by the over-expression of the mesenchymal markers YKL-40 and Vim [5,6,7,8]. We began investigating the relationship between the mGBM signature, tumor vascularization, and immune checkpoint protein levels. By means of the public available datasets of differential gene profiles established with large human cancer cohorts (http://gepia.cancer-pku.cn, accessed on 1 January 2018), we found that, out of 11 human cancers, GBM was among the top tumors displaying the greatest elevation in YKL-40 expression relative to the corresponding controls (Figure S1A). GBMs also expressed the highest transcript level of YKL-40 in an analysis of 33 human cancers (Figure S1B). We then evaluated the levels of YKL-40 expression in the four subsets of GBM and found that the mesenchymal type expressed the highest levels of YKL-40 (Figure 1A). In GBMs, YKL-40 and Vim expression levels were higher than in adjacent benign tissue (Figure 1B), and each marker was negatively associated with patient survival (Figure 1C). The expression of YKL-40 was positively correlated with Vim expression (Figure 1D). Consistent with previous reports that GBM expresses high levels of PD-L1 [35,36,37], the transcriptomic analysis demonstrated that the level of PD-L1 in mGBM was noticeably higher than in benign tissue, while GBM and the other GBM subtypes displayed a tendency for increased levels of PD-L1 relative to the corresponding controls (Figure 1E). Accordingly, elevated PD-L1 expression was correlated significantly with decreased patient survival for mGBM, but not for the other subtypes (Figure 1F). Indeed, YKL-40 and Vim were strongly correlated with PD-L1 expression (*p* = 4.4 × 10^−12^ and 1.9 × 10^−15^), whereas the correlation of the macrophage marker CD68 with PD-L1 levels was weaker (*p* = 1.7 × 10^−5^) (Figure 1G), suggesting that mesenchymal tumor cells rather than macrophages are keenly associated with PD-L1 expression. Next, we recruited a cohort of 38 GBM patients to independently validate these findings. Twenty-six out of thirty-eight cases (68.4%) were classified with mGBMs that displayed the co-expression of YKL-40 and Vim (Table 1). An IHC analysis demonstrated strong expression of YKL-40, Vim, PD-L1, and a vascular marker, VE-cad, in mGBM (Figure 1H). Interestingly, in the mGBM tumors, a significant cell population (64%) positive for PD-L1 was localized to vascular cells that co-expressed PD-L1 and CD31, an endothelial cell marker (Figure 1I). In the non-mGBM tumors, only 33% of the PD-L1-positive cells were endothelial cells (Figure S1C). In addition, PD-L1 levels were positively associated with tumor size (Figure S1D). Due to the limited number of cases in our study, the relationship between YKL-40 and PD-L1 or disease-free survival was insufficient to yield significant correlations (Table 1). Taken together, the data in the public datasets with large GBM cohorts suggest that mGBMs co-express high levels of YKL-40, Vim and PD-L1, which are correlated with each other and with a poor disease prognosis. In mGBM, a significant cellular population that is positive for PD-L1 is vascular endothelial cells, implicating this cell type role in vascular immune evasion.

### 3.2. YKL-40 Over-Expressed by GL261 Cells Induces PD-L1 + Vessel Angiogenesis and Restricts CTL Infiltration In Vivo

**Figure 2:**
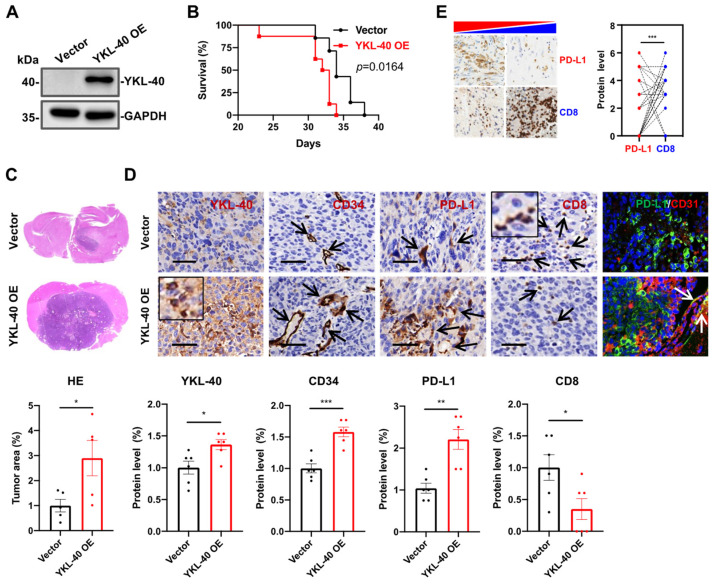
YKL-40 mediates the restriction of CD8+ cell infiltration and the induction of tumor development in GBM mouse models. (**A**) WB analyses of YKL-40 levels in GL261 cells expressing the control vector or YKL-40 (YKL-40 OE). (**B**) Survival of C57BL/6 mice receiving a brain transplant of vector control or YKL-40 OE GL261 cells (n = 6/group). (**C**) Hematoxylin and eosin (H&E) staining tissue sections. Data from a representative mouse for each group are shown. Scale bar, 200 μm. (**D**) Representative IHC and dual immunofluorescence staining for the indicated markers in mouse samples. The inserts show large YKL-40+ or CD8+ signals. The arrows indicate PD-L1+, CD34+ vessels, or CD8+ cells. Scale bar, 50 μm. These stains are quantified in the bottom panel (mean ± SEM, n = 5). (**E**) Representative images of PD-L1 and CD8 IHC staining in GBM patient samples (left) and quantification (right), Scale bar, 100 μm, n = 38. * *p* < 0.05, ** *p* < 0.01, *** *p* < 0.001. The uncropped blots are shown in File S1.

To determine if YKL-40-expressing tumors are associated with PD-L1-positive vessel development and decreased immune toxicity in vivo, we transplanted mouse glioma GL261 cells over-expressing the control vector or YKL-40 orthotopically into the brains of C57BL/6 mice (Figure 2A and Figure S2A). This model allowed us to monitor the effects of the YKL-40 protein on tumor development. Three weeks after transplantation, the mice displayed decreased locomotion. The survival of mice bearing YKL-40-expressing GL261 cells was significantly shorter than that of control mice that received vector control GL261 cells (Figure 2B). The brain tumors that developed in mice carrying YKL-40-expressing GL261 cells were approximately three-fold larger than those in control animals (Figure 2C). IHC analysis showed that the tumors expressing YKL-40 developed 50% more intense CD34-positive vessels but expressed 75% lower levels of CD8+ T cell infiltration than the control tumors (Figure 2D and bottom panel quantification). In addition, a larger number of vascular cells in YKL-40-expressing tumors were PD-L1-positive than those in control tumors by 2.2-fold, consistent with our earlier findings in mGBM tissue (Figure 1I). Dual immunofluorescence staining validated this result and showed the co-expression of PD-L1 and CD31 by vascular cells in YKL-40-expressing tumors (Figure 2D). These in vivo data suggest that YKL-40 expressed by tumor cells induces tumor angiogenesis and suppresses infiltrating CD8+ T cells, leading to more aggressive tumors and reduced animal survival. Then, we sought to determine if PD-L1 expression is negatively correlated with CD8+ T cells in GBM patient samples. In our cohort of GBM patients, we found that the elevated expression of PD-L1 was associated with lower levels of CD8+ cells, suggestive of a negative correlation (Figure 2E). Therefore, the outcome results with YKL-40-induced PD-L1 positive vascularization in the animal studies have potential benefits for patient diagnosis and prognosis.

### 3.3. YKL-40 Regulates PD-L1 through Activating the VE-Cad/β-Catenin/LEF Pathway in Endothelial Cells

**Figure 3:**
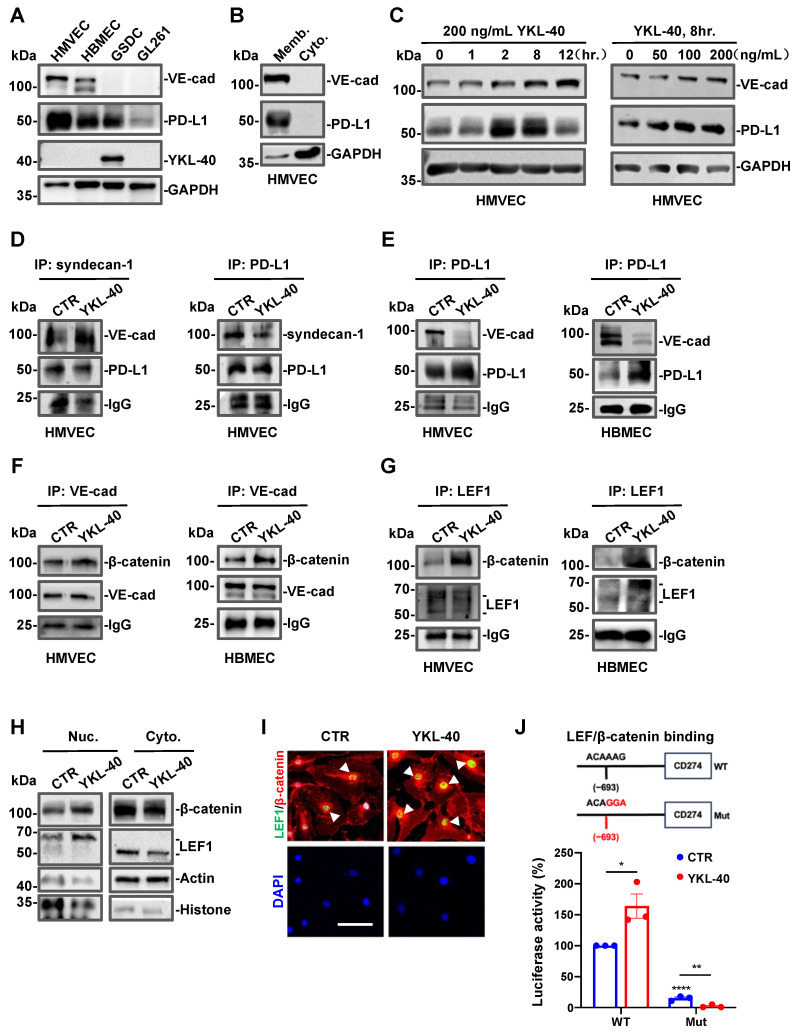
YKL-40 upregulates PD-L1 through activating the VE-cad/β-catenin/LEF pathway in vascular endothelial cells. (**A**) PD-L1, VE-cad, and YKL-40 expression in endothelial cell lines (HMVECs and HBMECs) and glioma cell lines (GSDC and GL261) by Western blot analysis. GAPDH expression was used as a loading control. (**B**) Subcellular locations of PD-L1 and VE-cad in HMVECs determined by immunoblotting. (**C**) HMVECs were incubated with recombinant YKL-40 protein (50, 100 or 200 ng/mL) for 1–12 h, and then cellular PD-L1 and VE-cad expression was analyzed. (**D**) Co-IP and WB analysis of the interaction of VE-cad/syndecan-1 and syndecan-1/PD-L1 (left), PD-L1/syndecan-1 (right) in HMVECs treated with recombinant YKL-40 protein (200 ng/mL). (**E–G**) Co-IP and WB analysis of the interaction between VE-cad/PD-L1 (**E**), β-catenin/VE-cad (**F**), and β-catenin/LEF1 (**G**) in HMVECs and HBMECs treated with YKL-40 (200 ng/mL). (**H,I**) Analysis of β-catenin and LEF1 subcellular locations in HMVECs following cell fractionation and immunoblotting (**H**) and immunofluorescence staining (**I**). The arrowheads indicate overlapped nuclear locations of LEF1 (green) and β-catenin (red). Scale bar, 100 μm. (**J**) Schematic representation of the CD274 promoter constructed into the pGL3-luciferase report vector. The β-catenin/LEF1 binding motif of WT and the mutant is shown at the top. The promoter activity of CD274 WT or mutant in 293T cells in the presence of YKL-40 was shown at the bottom (mean ± SEM, n = 3). * *p* < 0.05, ** *p* < 0.01, **** *p* < 0.0001. The uncropped blots are shown in File S1.

In order to validate that vascular tissue expresses high levels of PD-L1, we analyzed several cell lines, including human microvascular endothelial cells (HMVECs) and human brain microvascular endothelial cells (HBMECs), the human GBM tumor line GSDC, and the mouse line GL261. Both the HMVEC and HBMEC lines expressed VE-cad (Figure 3A). Consistent with earlier studies (Figure 1H,I), HMVECs, HBMECs, and GSDCs expressed high levels of PD-L1 compared with GL261 cells, which served as a control (Figure 3A and Figure S2B), Among the lines tested, only GSDCs expressed YKL-40. As expected, PD-L1 in HMVECs was restricted to the cell membrane fraction, comparable to the vascular membrane adhesion receptor VE-cad (Figure 3B). To determine if YKL-40 regulates PD-L1 on endothelial cells, we stimulated HMVECs with recombinant YKL-40 protein and found that YKL-40 induced PD-L1 expression in a time and dose-dependent manner (Figure 3C and Figure S2C). Interestingly, YKL-40 treatment also led to the upregulated expression of VE-cad. To investigate the regulatory activity of YKL-40 derived from tumor cells on vascular PD-L1 expression, we employed shRNA of YKL-40 gene knockdown in GSDCs (Figure S2D). We transferred GSDC conditioned media (CM) to HMVECs. CM from YKL-40 gene knockdown cells suppressed PD-L1 expression by 50% and suppressed VE-cad modestly relative to that seen with control CM. This unexpected partial effect may be ascribed to unknown factors present in CM that are regulated by YKL-40 gene knockdown. In our previous studies, we demonstrated that YKL-40 can bind to the ectodomain of syndecan-1 on HMVECs and regulate vascular angiogenesis [9,17]. Thus, we speculate that YKL-40 binds to syndecan-1 which, in turn, interacts with PD-L1 and/or VE-cad to activate an intracellular signaling cascade that leads to the upregulation of PD-L1 gene expression. To test this hypothesis, we first examined the interaction between syndecan-1 and VE-cad and PD-L1. Co-immunoprecipitation (Co-IP) analyses showed that YKL-40 promoted the binding of VE-cad to syndecan-1 but decreased the interaction between PD-L1 and syndecan-1 (Figure 3D). The reciprocal Co-IP results using an anti-PD-L1 antibody for the pulldown validated the decreased PD-L1/syndecan-1. Of note, YKL-40 also blocked the interaction of PD-L1 with VE-cad (Figure 3E and Figure S2E). These data support the model whereby YKL-40 binds to syndecan-1, which then recruits VE-cad, leading to the dissociation of PD-L1 from the complex of syndecan-1 and VE-cad.

To validate the active role played by mGBM-derived YKL-40, we determined the mesenchymal characteristics of GSDCs compared with GL261 cells as non-mesenchymal brain tumor cells. As shown in Figure S2F, in contrast with GSDCs, GL261 cells did not display a fibroblastic phenotype, nor did they express YKL-40, Vim, or smooth muscle alpha actin. The CM from GL261 cells induced a greater interaction between VE-cad and PD-L1 in HBMECs than CM from GSDCs. This supports our earlier findings that YKL-40 derived from mGBM decreases VE-cad and PD-L1 interactions on endothelial cells. 

Next, in order to investigate the intracellular signaling pathway regulated by VE-cad, we examined the association of VE-cad and β-catenin and found that YKL-40 induced their interaction (Figure 3F). Published studies have demonstrated that β-catenin and LEF can form a complex that translocate to the nucleus and drives target gene expression [38]. Consequently, we evaluated their interaction in endothelial cells and found that the treatment of HMVECs with YKL-40 induced an association between β-catenin and LEF1 (Figure 3G). This led to increases in the nuclear levels of β-catenin and LEF1 and concurrent reductions in the cytoplasm levels of these proteins (Figure 3H and Figure S2G). Dual immunofluorescence staining validated their translocation into the nucleus in the presence of YKL-40 (Figure 3I). To further determine if VE-cad-induced intracellular signaling regulates PD-L1 expression, we constructed a luciferase reporter gene driven by the CD274 promoter containing either the WT or mutant β-catenin/LEF binding sequence (Figure 3J). When the consensus binding site ACAAAG 693 bp upstream of the PD-L1 start code sequence was mutated to ACAGGA, YKL-40-induced luciferase reporter activity in WT 293T cells was eliminated in the mutant cells independently of YKL-40 stimulation. Even in the absence of VE-cad, 293T cells expressed E-cad capable of triggering similar intracellular activation of β-catenin/LEF via E-cad interaction with syndecan-1 (Figure S2H). These data demonstrate that YKL-40 induces the expression of PD-L1 through an interaction between syndecan-1 and VE-cad, and the subsequent transactivation of downstream β-catenin/LEF1.

### 3.4. Physical Interactions between PD-L1 and VE-Cad or PD-1

**Figure 4:**
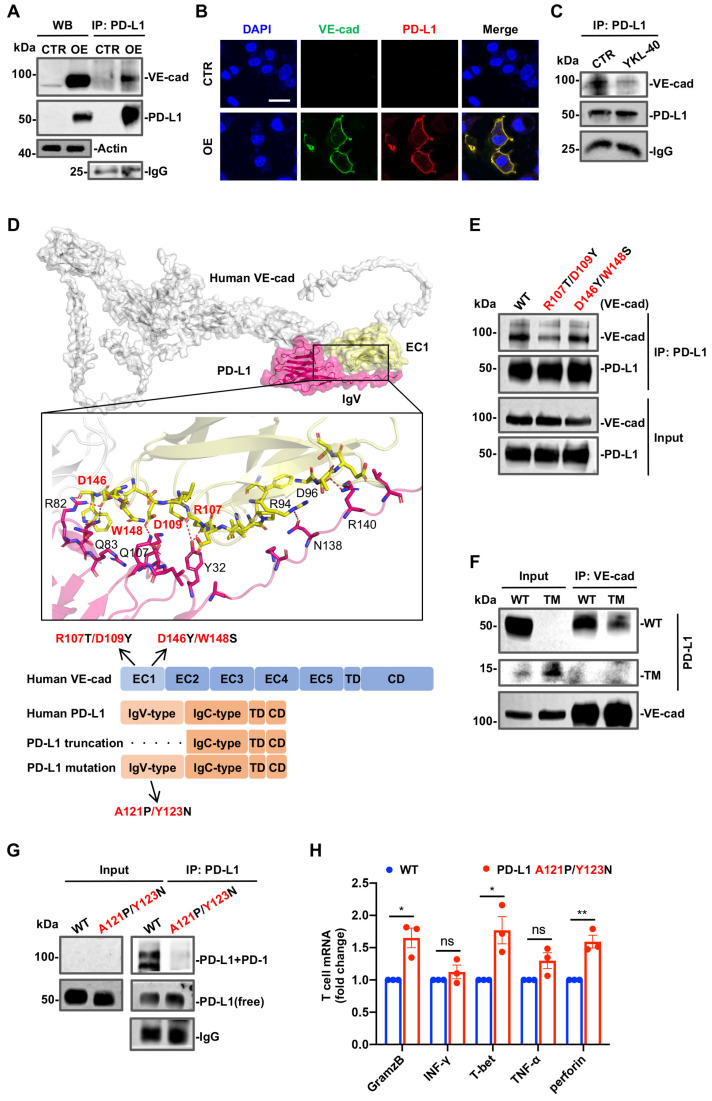
Molecular mechanism of the PD-L1 and VE-cad interaction. (**A**,**B**) 293T cells were engineered to stably express PD-L1 and VE-cad expression (OE) or acted as negative controls (CTR) and were analyzed for PD-L1 and VE-cad interactions by Co-IP and WB (**A**) and immunofluorescence (**B**). Scale bar, 25 μm. (**C**) Co-IP and WB analysis of the binding between PD-L1 and VE-cad OE 293T cells in the presence or absence of YKL-40. (**D**) Top: the modeled complex structure of PD-L1-VE-cad generated with the ClusPro program. The EC1 domain of VE-cad (yellow) appeared to interact with PD-L1 (pink). Middle: close-up view of the putative binding interface between VE-cad (EC1) and PD-L1 (IgV). Residues positioned at the interface are labeled as sticks, and hydrogen bonds are depicted as red, dashed lines. Labeled residues of each protein were hypothesized to interact with each other, in which red labeled sites of VE-cad were subsequently mutated. Bottom: schematic view of the VE-cad and PD-L1 gene map including each WT and mutants that were stably expressed in 293T cells. (**E**,**F**) Co-IP and WB analysis of the interactions between PD-L1 and VE-cad mutants (**E**) or PD-L1 truncated mutants (TM) (**F**) in 293T cells. (**G**) Co-IP and WB analysis for the interaction between PD-L1 and PD-1. TALL-104 cells (1 × 10^6^) were co-cultured with 293T cells (1 × 10^6^) expressing PD-L1 WT or the A121P/Y123N mutant. After 12 h, 4% paraformaldehyde was added to the cultures to induce protein–protein cross-linking. Cell lysates were subjected to Co-IP with the anti-PD-L1 antibody followed by immunoblotting with an anti-PD-1 antibody (top panel), an anti-PD-L1 antibody (middle panel), and IgG (bottom panel). (**H**) The same cell co-culture from (**G**) was used. Following the collection of suspension TALL-104 cells, these cells were used to determine immune factor expression via qPCR (mean ± SEM, n = 3). ns *p* > 0.05, * *p* < 0.05, ** *p* < 0.01. The uncropped blots are shown in File S1.

Our initial finding of a physical interaction between VE-cad and PD-L1 at the cell membrane encouraged us to define where the physical points of contact between the two proteins are. To minimize the possible influences of endogenous VE-cad and PD-L1 at the endothelial cell membrane and to enhance the efficiency of exogenous gene transduction into cells, we selected 293T cells, because they lack endogenous VE-cad and PD-L1 (Figure 4A). Since the cell adhesion of VE-cad is characterized by the cell adhesion recognition site (CAR) in the EC1 domain that is composed of the triple amino acid sequence HAV [39], we focused specifically on the EC1 region as the potential binding site for PD-L1. An expression construct encoding the wild-type ectodomain and transmembrane domain of VE-cad and one encoding a wild type of PD-L1 were created, and both were stably transduced into 293T cells (Figure 4A). Consistent with our earlier data, in the absence of YKL-40, VE-cad was physically associated with PD-L1 and localized exclusively at the cell membrane (Figure 4B). When the cells were stimulated with YKL-40, this association was interrupted (Figure 4C). In order to define the potential binding sites between VE-cad and PD-L1, we employed the ClusPro crystal structure of PD-L1 (PDB Code: 4ZQK) and the prediction of VE-cad crystal structure from AlphaFold 2 to simulate the PD-L1-VE-cad complex [40,41]. The predicted model revealed a polar protein–protein interface whereby R107, D109, D146, and W148 of VE-cad formed hydrogen bonds with the Y32, Q107, R82, and Q83 residues located at the solvent-exposed region of PD-L1 (Figure 4D). To test this model and validate their specific interaction, we created the mutants R107T/D109Y and D146Y/W148S constructs in which the basic amino acid arginine (R) was mutated to alcoholic threonine (T), acidic aspartate (D) was mutated to aromatic tyrosine (Y), and aromatic tryptophan (W) was mutated to alcoholic serine (S). We found that the R107T/D109Y point mutation in VE-cad remarkably reduced the ability of VE-cad to interact with PD-L1, while the D146Y/W148S point mutation did not have this impact (Figure 4E and Figure S3A). Furthermore, the docking analysis also suggests that the potential interaction region of PD-L1 is the IgV domain, which is known to bind to PD-1 (Figure 4D and Figure S3B). Consequently, we deleted the PD-L1 IgV region and found that this truncated version of the protein displayed a dramatic reduction in its association with VE-cad (Figure 4F). To further determine which site(s) in the IgV region are responsible for mediating the interaction of PD-L1 with PD-1 expressed by T cells, we generated the A121P/Y123N mutant in this IgV region. We then co-cultured wild-type or mutant 293T cells with TALL-104 cells, a CD8+ T cell line that is MHC non-restricted tumor-lytic, particularly for glioma [42,43,44,45]. We then treated the co-cultures with the irreversible cross-linking reagent paraformaldehyde in order to determine the physical association between PD-L1 and PD-1 in both cell types. Co-IP and Western blotting (WB) results demonstrated that the A121P/Y123N mutation protected the interaction of PD-L1 with PD-1, as the 100 kDa complex of PD-L1/PD-1 was observed in wild-type cells but not in the mutant lines (Figure 4G). Blockade of this interaction resulted in the elevated expression of immune factor proteins (e.g., GramzB, T-bet, Perforin) by TALL-104cells (Figure 4H). Altogether, our results provide mechanistic insight into the interaction model in which R107 and D109 in VE-cad respond to its interaction with PD-L1, while A121 and Y123 in PD-L1 contribute to its interaction with PD-1. These data also provide compelling evidence suggesting that PD-L1 may act as a central molecule to regulate angiogenesis and anti-immunity via interactions with either VE-cad or PD-1.

### 3.5. YKL-40 Promotes the Interaction between PD-L1 and PD-1, Which Drives Vascular Immune Evasion

**Figure 5:**
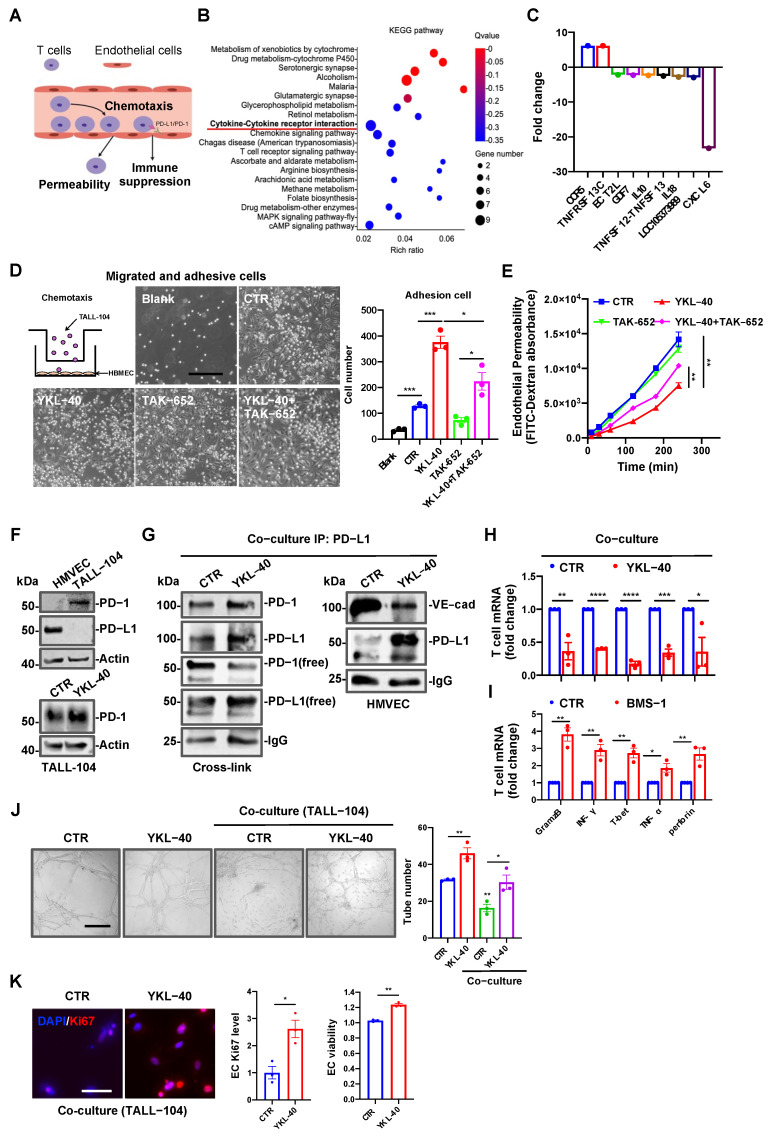
YKL-40 promotes CTL chemotaxis toward the endothelium but blocks CTL anti-vessel immunity. (**A**) A model of CTL chemotaxis toward HMVECs and infiltration. (**B**) RNA-seq analyses and KEGG pathway analysis in HMVECs after treatment with or without YKL-40. The red line highlights the cytokine–cytokine receptor interaction pathway. (**C**) The candidate genes of the cytokine–cytokine receptor interaction pathway (fold change > 2), n = 3. (**D**) The picture shows the experimental strategy used to test TALL-104 cell chemotaxis and adhesion. HBMECs (5 × 10^4^) were grown to be confluent in the bottom chamber of 8 μm transwells, and then TALL-104 cells (1 × 10^6^) were added to the top chamber. Serum-free cell medium was introduced to both chambers, and YKL-40 (200 ng/mL) or TAK-652 (30 nM) was added to the bottom chamber. After 12 h, after suspended TALL-104 cells at the bottom chamber had been removed, the remaining TALL-104 cells adhering to HBMECs were imaged and analyzed. Scale bar, 50 μm, blank: no HBMECs (mean ± SEM, n = 3). * *p* < 0.05, *** *p* < 0.001. (**E**) HMVECs (1 × 10^5^) were loaded onto the top chamber of 0.4 μm transwells. After confluence, the cells were treated with YKL-40 (200 ng/mL) or TAK-652 (30 nM) for 8 h. The cellular permeability was evaluated by measuring the fluorescence of FITC–Dextran at the bottom over 4 h after adding it to the top chamber (mean ± SEM, n = 3). ** *p* < 0.01. (**F**) PD-L1 and PD-1 expression in HMVECs and TALL-104 cells (top); PD-1 expression in TALL-104 cells treated with or without YKL-40 (200 ng/mL) (bottom). (**G**) Co-IP and WB analysis of the interaction between PD-L1 (HMVECs) and PD-1 (TALL-104 cells) after co-culture in the presence of YKL-40 (200 ng/mL) and cross-linking using 4% paraformaldehyde for 10 min (left), and the interaction between PD-L1 and VE-cad in HMVECs after co-culture (right). (**H, I**) RT-qPCR qualification of the cytotoxic factor mRNAs produced by TALL-104 cells after co-culture, as in (**G**). (**H**): Pre-treated with YKL-40 (200 ng/mL); (**I**): co-cultured with BMS-1 (1 μM), an inhibitor of the PD-L1/PD-1 interaction (mean ± SEM, n = 3). * *p* < 0.05, ** *p* < 0.01, *** *p* < 0.001, **** *p* < 0.0001. (**J**) Tubes induced by HMVECs co-cultured with TALL-104 cells (1:1) in the presence of YKL-40 (200 ng/mL). Images of tubes (left) and quantification (right) (mean ± SEM, n = 3). * *p* < 0.05, ** *p* < 0.01. Scale bar, 50 μm. (**K**) The proliferation of HBMECs co-cultured with TALL-104 cells (1:1) in the presence of YKL-40 (200 ng/mL) was evaluated by immunocytochemistry staining with an anti-Ki67 antibody (red). Scale bar, 50 μm. Likewise, the survival of HBMECs was determined using a CCK-8 kit (mean ± SEM, n = 3). * *p* < 0.05, ** *p* < 0.01. The uncropped blots are shown in File S1.

The observation that VE-cad induced the expression of PD-L1 encouraged us to test the hypothesis that elevated PD-L1 expression enhances the interaction between PD-L1 and PD-1 which, in turn, inhibits CTL-mediated anti-vascular immunity. To test this hypothesis, we mimicked an in vivo model of circulatory leukocyte infiltration into tumors and examined the interaction of infiltrating T cells with tumor cells using transwell cell migration and adhesion assays (Figure 5A). First, to explore the recruitment of CTLs to YKL-40-activated endothelial cells in vessels, we performed a transcriptomic analysis (RNA-seq) of HMVECs treated with YKL-40. The KEGG analysis revealed that YKL-40 significantly induced the expression of cytokine–cytokine receptor signaling pathways (Figure 5B), in which CCR5-mediated signaling was one of the two notable upregulated pathways, whereas seven other components were downregulated (Figure 5C). To evaluate the role of CCR5 in chemotaxis and adhesion, we grew HBMECs in the bottom chamber of transwells and loaded TALL-104 cells expressing CCL5 (Figure S4A) into the top chamber in the presence of either YKL-40 or the CCR5 inhibitor TAK-652 overnight. Following the removal of TALL-104 suspension cells from the bottom chamber, the adhesion of TALL-10 cells to HBMECs was analyzed (Figure 5D). YKL-40 promoted the adhesion of TALL-104 cells to HBMECs by 2.2-fold relative to controls. In contrast, TAK-652 reduced their adhesion by 40% relative to YKL-40-induced cells. The direct addition of TALL-104 cells to HMVECs in a monolayer culture resulted in identical TALL-104 cell adhesion in the presence of YKL-40 and/or TAK-652 (Figure S4B). Next, we assessed the impacts of YKL-40 on HMVEC permeability by loading Dextran into the top chamber that pre-grew TALL-104 cells and measured the permeable Dextran in the lower chamber. YKL-40-treated HMVECs exhibited lower permeability than controls, whereas TAK-652 resulted in a partial rescue (Figure 5E).

We next tested the hypothesis that YKL-40 restricts endothelial cell permeability leading to the accumulation of CTLs in the vessels, which facilitates the interaction of PD-L1 with PD-1. We measured the basal levels of PD-L1 and PD-1 expressed in HMVECs and TALL-104 cells, respectively (Figure 5F). Like PD-L1, YKL-40 also induced the expression of PD-1 in TALL-104 cells (Figure 5F). To evaluate the physical interaction between PD-L1 and PD-1, we set up the co-culture system with both cells as before (Figure 4G) and then added YKL-40. We found that YKL-40 prompted the association of PD-L1 with PD-1, as the 100 kDa complex of PD-L1/PD-1 was observed during immunoblotting using either an antibody against PD-L1 or PD-1 (Figure 5G and Figure S4C). In a separate experiment, we collected HMVECs and TALL-104 cells individually after treatment with YKL-40 in the co-culture system. In agreement with our earlier results (Figure 3E), YKL-40 treatment resulted in the disassociation of PD-L1 from VE-cad and simultaneously induced the expression of PD-L1 (Figure 5G). We employed qPCR to measure the expression of immune active factors in the co-cultured TALL-104 cells. As shown in Figure 5H, the exposure of TALL-104 cells to YKL-40 led to marked suppression of GramzB, INF-γ, T-bet, TNF-α, and perforin expression by 60–75% relative to the controls. The TALL-104 cell number and viability were also significantly decreased in this co-culture system (Figure S4D). To verify the specific function of PD-L1/PD-1 in co-cultures, we added BMS-1, a specific inhibitor of the PD-L1/PD-1 interaction. BMS-1 treatment increased the expression of immune factors in TALL-104 cells (Figure 5I). Finally, we tested the functional effects of TALL-104 cells on HMVECs. YKL-40, a well-known pro-angiogenic factor, induced HMVEC tube formation (Figure 5J). However, when TALL-104 cells were incubated with HMVECs, tube formation was inhibited by ~50%. The ability of YKL-40 to induce tubes was also reduced to the basal level of control cells in the presence of TALL-104 cells (Figure 5J). Likewise, we also found that YKL-40 induced endothelial cell proliferation and viability compared with no treatment in the co-culture with TALL-104 cells (Figure 5K). Taken together, these data suggest that YKL-40 drives CTL chemotaxis to endothelial cells through the CCL5/CCR5 interaction and stimulates the disassociation of VE-cad from PD-L1, which interacts with PD-1 on TALL-104 cells, leading to vascular cell escape from CTL immunity.

### 3.6. VE-Cad and PD-L1 shRNA in HMVECs Restore Cytotoxicity of CTLs

**Figure 6:**
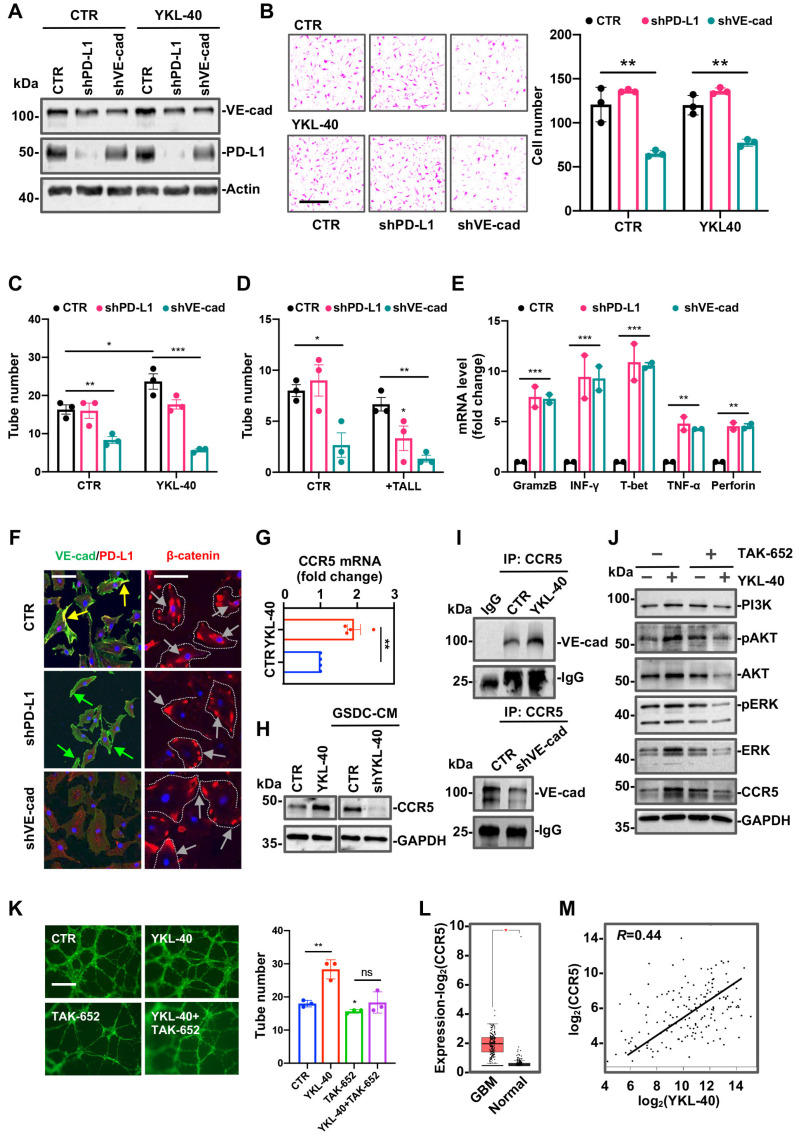
VE-cad and PD-L1 gene knockdown decreases vascularization in response to CTLs, and the interaction of VE-cad with CCR5 mediates YKL-40-induced angiogenesis. (**A**) HMVECs were engineered to stably express shPD-L1, shVE-cad, or CTR shRNA and then were treated with YKL-40 (200 ng/mL) for 8 h. Cellular PD-L1 and VE-cad expression were analyzed by immunoblotting. (**B**) Cell migration was analyzed in these engineered cells using transwells. Images of migrated cells (left) and quantification (right) (mean ± SEM, n = 3). ** *p* < 0.01. Scale bar, 50 μm. (**C**) The cells in (**A**) were analyzed for tube formation (mean ± SEM, n = 3). * *p* < 0.05, ** *p* < 0.01, *** *p* < 0.001. (**D**) The cells in (**A**) were mixed with TALL-104 cells (1:1) for the tube formation assay (mean ± SEM, n = 3). * *p* < 0.05, ** *p* < 0.01. (**E**) These cells in A were co-cultured with TALL-104 cells (1:1). After 12 h, removed TALL-104 cells were collected to measure the cytotoxic factor mRNA via RT-qPCR (mean ± SEM, n = 2). ** *p* < 0.01, *** *p* < 0.001. (**F**) Immunofluorescence analysis of PD-L1 (red)/VE-cad (green) and β-catenin (red) staining in HMVECs. Yellow arrows indicate the overlapping expression of PD-L1/VE-cad, the green arrows show VE-cad, and the gray ones indicate the locations of β-catenin. Note the cytoplasm location of β-catenin in CTR and its membrane association in shVE-cad and shPD-L1 cells. DAPI staining was blue. Scale bar, 100 μm. (**G**) HMVECs were incubated with or without YKL-40 (200 ng/mL) for 12 h, and then the CCR5 mRNA level was analyzed by RT-qPCR (mean ± SEM, n = 3). ** *p* < 0.01. (**H**) CCR5 protein levels in HMVECs treated with YKL-40 (200 ng/mL) or conditioned media from CTR or shYKL-40 cells were analyzed by immunoblotting. (**I**) The interaction of CCR5 with VE-cad in HMVECs treated with YKL-40 (200 ng/mL) was analyzed by Co-IP and WB (top). Decreased interaction of CCR5 with VE-cad in shVE-cad-expressing HMVECs (bottom). (**J**) HMVECs were incubated with YKL-40 (200 ng/mL), TAK-652 (30 nM), or their combination for 8 h. The Akt and MAPK pathways were analyzed by immunoblotting. (**K**) Analysis of tube formation by HMVECs in the presence of YKL-40 (200 ng/mL) or TAK-652 (30 nM). Images (left), Scale bar, 50 μm; quantification of tubes via counting intact circled tubes (right) (mean ± SEM, n = 3). ns: no significant, * *p* < 0.05, ** *p* < 0.01. (**L**,**M**) CCR5 mRNA level (**L**) and the correlation with mRNA YKL-40 (**M**) in GBM (normal = 163, tumor = 207) were analyzed from the database (http://gepia.cancer-pku.cn, accessed on 1 January 2018), * *p* < 0.05. The uncropped blots are shown in File S1.

To assess the roles of VE-cad and PD-L1 in endothelial cell angiogenesis resistance to CTL immunity, we performed VE-cad or PD-L1 gene knockdown in HMVECs. HMVECs cells were transfected with shVE-cad or shPD-L1 lentiviral DNA constructs in order to establish stable cell lines (Figure S5A,B). VE-cad shRNA decreased the expression levels of both VE-cad and PD-L1. In contrast, PD-L1 shRNA only inhibited the expression of PD-L1 and not VE-cad expression, which was independent of YKL-40 treatment (Figure 6A and Figure S5C). The results support the regulation of VE-cad on PD-L1 gene expression found earlier. VE-cad and PD-L1 shRNA also attenuated the effects of YKL-40 on PD-L1 and the VE-cad association (Figure S5D). VE-cad shRNA reduced the ability of HMVECs to migrate compared with control cells (Figure 6B). In contrast, PD-L1 shRNA did not impact the effect of HMVECs on motility. Likewise, VE-cad shRNA cells displayed reduced tube formation and also failed to respond to YKL-40 (Figure 6C and Figure S5E). To determine if these shRNA cells are sensitive to the immune cytotoxicity mediated by TALL-104 cells, we added TALL-104 cells to control and engineer PD-L1 or VE-cad shRNA HMVECs and found a 50–70% reduction in tube formation in each line relative to the controls (Figure 6D and Figure S5F). Concomitantly, these TALL-104 cells co-cultured with HMVECs expressing VE-cad or PD-L1 shRNA expressed 4~15-fold higher levels of immune cytotoxic factors than corresponding controls (Figure 6E). In a dual-immunofluorescence staining analysis, VE-cad shRNA cells exhibited lower levels of both VE-cad and PD-L1 than control cells, while PD-L1 shRNA cells only resulted in decreased PD-L1 expression (Figure 6F), consistent with our previous WB result (Figure 6A). Interestingly, while β-catenin was located in both the cell membrane and cytosol in the control cells, it was largely restricted to the membrane in both shRNA cell lines (Figure 6F). This result suggests that the intracellular dysfunction of β-catenin likely accounts for decreased PD-L1 gene expression. Taken together, the data generated with the co-culture system demonstrate that VE-cad acts as a central factor to interact with and drive the expression of PD-L1 which, in turn, inactivates CTL immunity via PD-L1/PD-1 coupling.

In an attempt to determine if YKL-40 facilitates the intracellular signaling cascade that mediates VE-cad and/or CCR5-associated angiogenesis, we treated HMVECs with YKL-40 and observed that it led to the induction of both the CCR5 mRNA and protein (Figure 6G,H). In contrast, CM from GSDCs expressing YKL-40 shRNA resulted in a decrease in CCR5 expression relative to that of control cells. Next, we found that YKL-40 promoted the association of CCR5 and VE-cad (Figure 6I). In contrast, VE-cad shRNA in HMVECs led to a decreased association of CCR5 with VE-cad (Figure 6I). It is known that CCR5 regulates multiple intracellular signaling cascades, including PI3K/Akt and MAPK [46,47]. We then exposed HMVECs to YKL-40 in the presence or absence of the CCR5 inhibitor TAK-652 (Figure 6J). YKL-40 induced the expression of PI3K, pAkt, Akt, Erk1/2, and pErk1/2 (Figure 6J and Figure S5G). The ability of YKL-40 to induce the expression of these signaling molecules was prevented by co-treatment with TAK-652, suggesting that CCR5-mediated PI3K/Akt and MAPK signaling contributes to YKL-40-mediated intracellular signaling activation. In functional analyses, we found that YKL-40-induced tube formation was fully abrogated by TAK-652 (Figure 6K), underscoring the notion that YKL-40 stimulates the interaction of VE-cad and CCR5 and thus facilitates PI3K/Akt and MAPK-mediated angiogenic signaling and vascular angiogenesis. Supporting our findings, the public database (http://gepia.cancer-pku.cn, accessed on 1 January 2018) revealed that GBM tissue expressed higher levels of CCR5 than normal controls (Figure 6L) and that CCR5 expression was correlated with YKL-40 expression (Figure 6M). Taken together, all of these data suggest that YKL-40 actively promotes tumor development and inhibits anti-tumor immunity via the induction of PD-L1-mediated tumor vascularization.

## 4. Discussion

**Figure 7:**
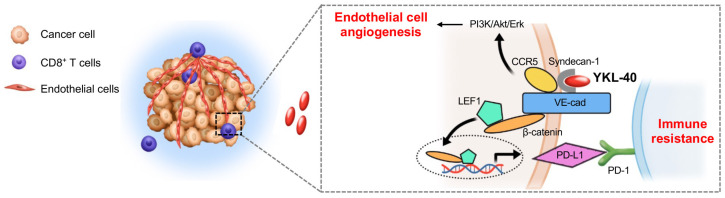
Schematic representation of YKL-40-induced angiogenesis and vascular immune evasion in mGBM.

A large body of clinical evidence has demonstrated that the upregulation of the immune checkpoint factor PD-L1 in GBM drives anti-tumor immunity [35,36,37,48]. Here, we unexpectedly discovered that PD-L1 is upregulated in vascular endothelial cells in mGBM, and this was correlated with the expression levels of YKL-40 and PD-L1. In agreement with these clinical data, our studies with the HMVECs and HBVECs displayed high levels of PD-L1 expression relative to macrophages, which expressed low levels of PD-L1. Although TAMs were reported to expresses PD-L1 [49,50], the correlation of TAM with PD-L1 compared with tumor cells (YKL-40 and Vim) was weak, and the contribution of PD-L1 expressed by TAMs to tumor vascularization in mGBM remains to be further investigated. Thus, our current data provide compelling evidence that PD-L1 expression is notably localized to endothelial cells, which motivated us to establish a model whereby PD-L1 induced by YKL-40 in endothelial cells renders the tumor vasculature resistant to CTL-mediated tumor immunity, thus facilitating tumor vessel-driven malignant transformation (Figure 7). Indeed, these PD-L1+-vascularized tumors inhibited infiltrating CD8+ CTLs in tumor xenografts. In line with this evidence, our in vitro co-culture systems revealed that YKL-40 restricted endothelial cell permeability, the vascular barrier that attenuates CTL infiltration and activity in tumors. In agreement with our findings, results from multiple clinical trials demonstrate that single anti-PD-L1 or anti-PD-1 antibodies confer only a transient benefit to patients, whereas when they were combined with anti-angiogenic blockers (e.g., bavecizumab), there was significantly longer patient survival [31,32]. Therefore, the current findings support the proof-of-principle that the combined regimen of anti-angiogenic agents with anti-immune checkpoint PD-L1 or PD-1 antibodies may offer promising benefits to patients with mGBM.

PD-L1 is a membrane receptor that is regulated by a number of growth factors (e.g., TGF-β), cytokines (e.g., INF-γ), and intracellular proteins (e.g., RAS, MAP) [51,52,53]. However, to date, its potential regulation by VE-cad has not been described. VE-cad, an endothelial marker, functions to regulate cell–cell adhesion, which mediates vascular formation [54,55]. Intriguingly, we found that VE-cad interacts with adjacent PD-L1 at the membrane where VE-cad regulates PD-L1 activity and triggers an intracellular signaling pathway to induce PD-L1 expression. R107 and D109 in the EC1 of VE-cad are core elements in the binding to PD-L1, since the point mutation R017T/D109Y resulted in the notable inhibition of the VE-cad interaction with PD-L1. Like VE-cad, mutated PD-L1 with a truncated extracellular IgV-domain lost the ability to interact with VE-cad, supporting the hypothesis that both ectodomain binding sites are essential for their interaction. While potentially distinct positions (e.g., Y56, E58, R113) of PD-L1 are known to participate in the interaction with PD-1 on T cells [56,57], our study with the A121P/Y123N mutant in the IgV region revealed the disruption of the interaction between PD-L1 and PD-1. We predict that the interaction of PD-L1 with VE-cad or PD-1 occurs in a non-competitive binding manner due to the presumably distinct binding sites on PD-L1. Nevertheless, the 3D interaction between PD-L1 and VE-cad or PD-1 is required to substantially characterize the structural feature accounting for their spatial interaction. Intriguingly, we found that endothelial cells treated with YKL-40 resulted in the dissociation of VE-cad from PD-L1, rendering PD-L1 highly accessible to PD-1 and resulting in impaired immune cytotoxicity. Concurrently, the increased association of VE-cad with syndecan-1 induced the intracellular interaction of β-catenin with LEF, which then translocated to the nucleus and triggered PD-L1 expression, offering a positive feedback loop for the PD-L1/PD-1 interaction. Therefore, our study shows that VE-cad serves as an indispensable factor that mediates YKL-40-induced vascular immune escape. 

The trans-endothelial cell migration in vessels is rate-limiting for the leukocyte infiltration, which typically involves three sequential steps such as cell rolling, adhesion and spreading, and transmigration [58]. Our study found that YKL-40 enhanced the CCR5-mediated chemotaxis of CTLs towards endothelial cells. However, the subsequent cellular adhesion between CTLs and endothelial cells led to CTL dysfunction and accumulation at the vascular lumen, where YKL-40 restricted the vascular permeability and impaired CTL cytotoxicity via PD-1/PD-L1 coupling. Concomitantly, there is an enhanced interaction between CCR5 and VE-cad mediated by YKL-40 on endothelial cells, which helps to drive tumor angiogenesis. In agreement with published reports [46,47], our data demonstrate that activated CCR5 induces the PI3K/AKT and ErK intracellular signaling pathways which, in turn, mediates tumor vascularization. Thus, VE-cad plays dual key roles in the regulation of tumor angiogenesis and immune cell evasion in mGBM (Figure 7). 

The biggest limitation of this study is the lack of the establishment of mGBM cells in animals, which can fully reflect the signature of the YKL-40-induced PD-L1-expressing vasculature that resists anti-tumor immunity in vivo. Here, we employed mouse-derived glioma cells GL-261 that were enforced to express YKL-40, mimicking the mGBM function in mice. Although the results support our conclusion, the model is insufficient to entirely represent the genetic and functional characteristics of mGBM that remain to be fully deciphered. The optimal model at present is to use fully humanized mice transplanted with GSDCs. However, these costly animals are beyond the currently affordable budget for this study.

YKL-40 functions as a pro-angiogenic factor to promote tumor angiogenesis via interacting with syndecan-1 or IL-13R2α. Moreover, evidence from clinical trials employing the YKL-40 receptor IL-13R2α-targeting CAR T cells in patients with GBM demonstrated a dramatic restriction in tumor progression but increased recruitment of immune cells and multiple immune factors [59,60]. In line with these therapeutic studies, our in vivo study provides strong evidence that the gene silencing of YKL-40 in tumor cells remarkably suppresses tumorigenesis, highlighting a novel and promising therapeutic strategy tailored to the abrogation of angiogenesis and the reinvigoration of anti-tumor immunity.

## 5. Conclusions

Our study sheds light on a novel mechanism of immune evasion in the mesenchymal subtype of glioblastoma (mGBM), which is characterized by aggressive vasculature. Through clinical analysis and experimental investigations, we identified a mechanistic link between tumor vascularization and resistance to anti-tumor immune therapy. Our findings reveal that elevated expression of the mesenchymal markers YKL-40 and Vimentin in GBM is associated with increased expression of PD-L1 and poor disease survival. Remarkably, PD-L1 expression was predominantly observed in vascular endothelial cells. Further experiments using glioma cells over-expressing YKL-40 in mice demonstrated enhanced angiogenesis, reduced CD8+ T cell infiltration, and decreased survival rates. We discovered that exposure to the recombinant YKL-40 protein induced PD-L1 and VE-cadherin expression in endothelial cells, leading to the nuclear translocation of β-catenin/LEF and the subsequent upregulation of PD-L1 expression. YKL-40 promoted the dissociation of VE-cadherin from PD-L1, enabling PD-L1 to interact with PD-1 on CD8+-positive TALL-104 lymphocytes and inhibit their cytotoxicity. Additionally, YKL-40 facilitated TALL-104 cell migration and adhesion to endothelial cells via CCR5-dependent chemotaxis while impairing their anti-vascular immunity. However, the knockdown of VE-cadherin or PD-L1 genes abolished the effects of YKL-40 and rejuvenated TALL-104 cell immunity against the tumor vasculature. Collectively, our study unravels a previously unknown vascular immune escape mechanism employed by mGBM to promote tumor vascularization and drive malignant transformation. These findings provide valuable insights into the development of targeted therapies aimed at overcoming immune resistance in this aggressive subtype of glioblastoma with the potential to improve patient outcomes and survival.

## Figures and Tables

**Table 1:** Pathologic characteristics of GBM patients.

Case No.	Age	Sex	Protein Levels ^a^					DFS ^b^ (Months)	Tumor Size (mm)
			YKL-40	Vimentin	PD-L1	VE-Cad	CD8		
1	69	Male	5	4.5	0	3	0	5	28 × 35
2	53	Female	5	2.5	0	0	0	9	40 × 51 × 39
3	72	Male	5	0	5	4	3	2	26 × 34
4	59	Male	5	4.5	3	5	3	8	62 × 30
5	69	Female	3.5	4	0	4	0	6	35 × 39 × 48
6	46	Female	5	6	0	3	5	10	50 × 44
7	33	Male	3.5	3	0	4	ND	41	2.5
8	52	Male	5	ND	4	2	0	11	47 × 41 × 34
9	60	Male	2.5	3	0	2	3	9	42 × 60
10	53	Male	2.5	2.5	0	3	2	32	57.5 × 50
11	47	Male	5	5	5	4	0	10	45 × 15 × 21
12	57	Female	3	3.5	0	6	4	7	33 × 34 × 34
13	40	Male	3.5	3.5	0	4	0	4	29 × 53
14	51	Male	4	5	0	2	0	2	38 × 43 × 55
15	35	Male	2.5	4.5	0	0	0	>48	47 × 45 × 50
16	51	Male	5	4	0	2	4	5	44.5 × 40.9 × 54.9
17	52	Male	4	5.5	5	5	5	8	65 × 43
18	40	Female	2	5.5	3	2	4	>19	21 × 25 × 30
19	47	Male	4.5	5.5	0	5	5	8	26 × 33
20	44	Male	ND ^d^	ND	2	3	3	5	40 × 55
21	62	Female	3.5	6	2	3	5	3	48 × 48 × 38
22	48	Female	5	5	2	4	4	NA ^e^	29 × 19
23	66	Male	3	5.5	2	4	4	NA	47 × 67
24	43	Male	5.5	5.5	6	4	3	NA	50 × 40
25	71	Female	5	4.5	0	5	0	3.5	35 × 50
26	62	Male	4.5	4.5	0	6	3	NA	33 × 30
27	78	Male	2	3	0	5	5	1	24 × 19
28	62	Female	3.5	4	5	3	4	3	47 × 51 × 46
29	35	Female	2	3	0	6	5	NA	28 × 37
30	56	Male	3	3	4	6	3	NA	34 × 51 × 47
31	65	Male	3.5	3.5	0	4	5	NA	6 × 5 × 4
32	60	Male	4.5	5.5	0	4	6	1	47 × 41 × 32
33	62	Female	1	3.5	0	3	4	NA	33 × 41 × 32
34	46	Male	4	5.5	0	4	3	3	69 × 51
35	51	Female	1	3	3	0	ND	NA	38 × 37 × 43
36	76	Male	1	3	0	4	0	5	49 × 30
37	53	Female	1	5	0	3	3	NA	25 × 33 × 35
38	51	Male	2.5	3	0	3	4	NA	17.9
Total 38	Mean 54.6	F/M ^c^ 13/25	P/N 26/11	P/N 33/3	P/N 10/28	P/N 30/8	P/N 25/11		

^a^: semi-quantification, weak: 0–2.5, moderate: 3–4, high: 5–6 points. Negative (N): 0–2.5, positive (P): 3–6 points. ^b^: disease-free survival. ^c^: F/M female/male. ^d^: Not determined. ^e^: Not available.

## Data Availability

The data can be shared upon request.

## Supplementary Materials

The following supporting information can be downloaded at: https://www.mdpi.com/article/10.3390/cancers15174257/s1, Figure S1. Expression of YKL-40 and PD-L1 expression in human cancers. Figure S2. YKL-40 upregulates PD-L1 through activation of the VE-cad/β-catenin/LEF pathway in endothelial cells. Figure S3. VE-cad and PD-1 bind to the same region of PD-L1. Figure S4. Cell adhesion and the interaction between endothelial cells and TALL-104 cells are mediated by interaction of PD-1/PD-L1. Figure S5. shPD-L1 and shVE-cad in HMVECs restores the cytotoxic activity of CTLs. File S1: Western blot information.
