# Supplementary material for: Vascular Immune Evasion of Mesenchymal Glioblastoma Is Mediated by Interaction and Regulation of VE-Cadherin on PD-L1
Source: Cancers (Basel). 2023 Aug 25;15(17):4257. doi: 10.3390/cancers15174257 (PMC10486786; doi:10.3390/cancers15174257)

# Vascular Immune Evasion of Mesenchymal Glioblastoma Is Mediated by Interaction and Regulation of VE-Cadherin on PD-L1

WB analyses of YKL-40 levels in GL261 cells expressing control vector or YKL-40 (YKL-40 OE).

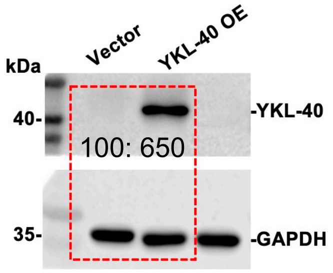


PD-L1, VE-cad and YKL-40 expression in endothelial cell lines (HMVECs and HBMECs) and glioma cell lines (GSDC and GL261) by Western blot analysis

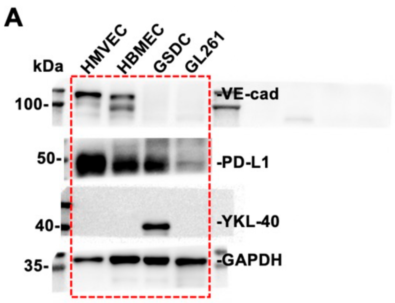


100: 60: 3: 1 VE-cad

100: 41: 18: 15 PD-L1

100: 160: 9738: 77 YKL-40

Subcellular location of PD-L1 and VE-cad in HMVECs by immunoblotting

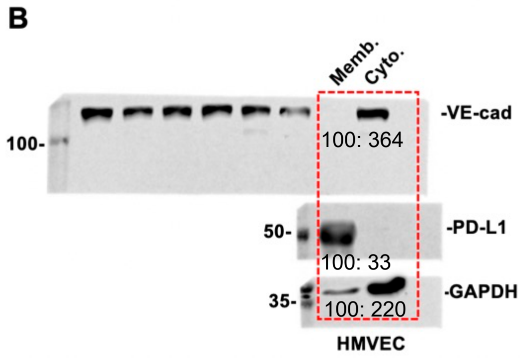


HMVECs were incubated with recombinant YKL-40 protein (50, 100 or 200 ng/mL) for 1-12 hours and then cellular PD-L1 and VE-cad expression were analyzed

C

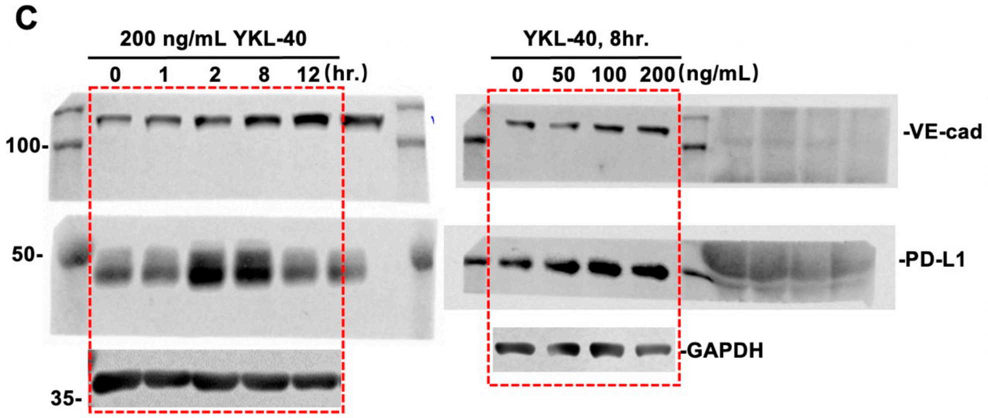


100: 86: 105: 121: 145 | 100: 103: 107: 134 | VE-cad

100: 184: 254: 281: 186 | 100: 140: 165: 325 | PD-L1

Co-IP and WB analysis of the interaction of VE-cad/syndecan-1 and syndecan-1/PD-L1 (left), PD-L1/syndecan-1 (right) in HMVECs treated with recombinant YKL-40 protein (200 ng/mL)

D

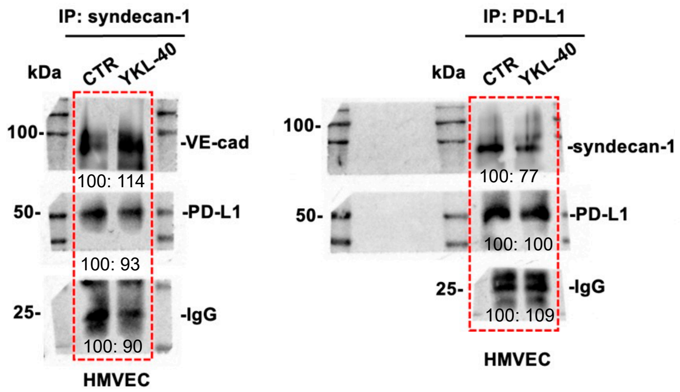


Co-IP and WB analysis of the interaction between VE-cad/PD-L1 (E), β-catenin/VE-cad (F) in HMVECs and HBMECs treated with YKL-40 (200 ng/mL)

E

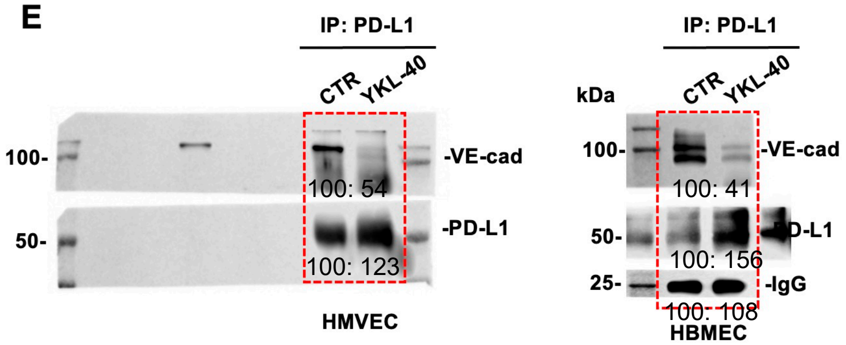

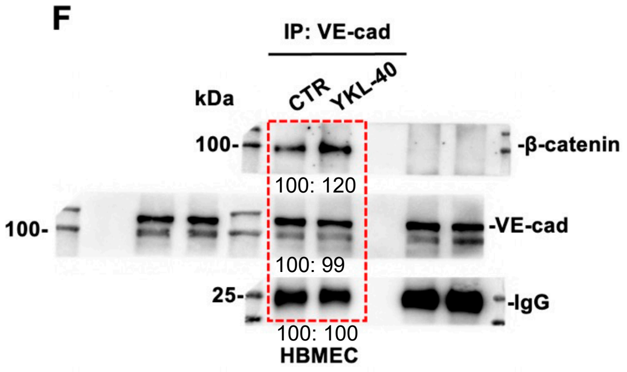


Analysis of β-catenin and LEF1 subcellular location in HMVECs following cell fractionation and immunoblotting

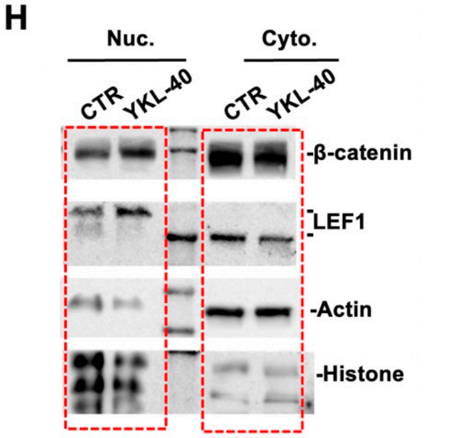


| | | |
|---|---|---|
| 100: 136 | 100: 64 | B-catenin |
| 100: 198 | 100: 85 | LEF1 |

293T cells were engineered to stably express PD-L1 and VE-cad expression (OE) or negative control (CTR) and analyzed for PD-L1 and VE-cad interaction by Co-IP and WB

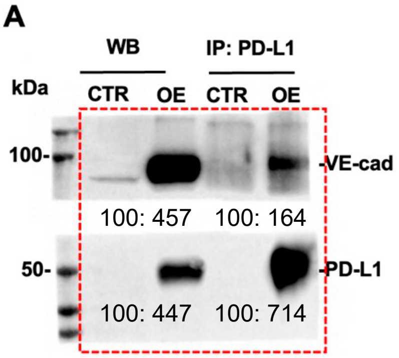


Co-IP and WB analysis of the binding between PD-L1 and VE-cad OE 293T cells in the presence or absence of YKL-40.

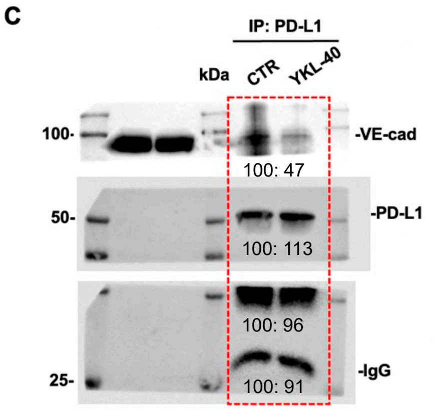


Co-IP and WB analysis of the interaction between PD-L1 and VE-cad mutants (E) or PD-L1 truncated mutants (TM) (F) in 293T cells.

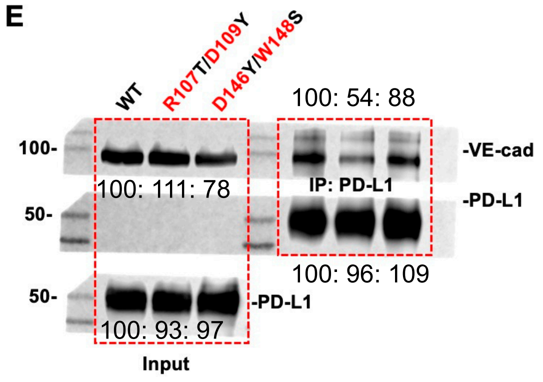


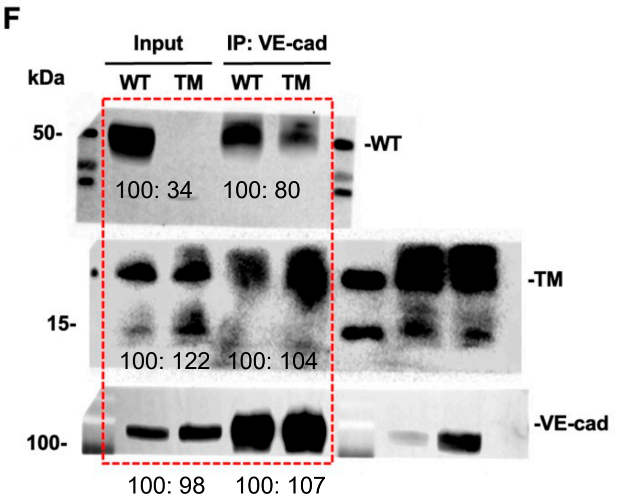


Co-IP and WB analysis for the interaction between PD-L1 and PD-1.

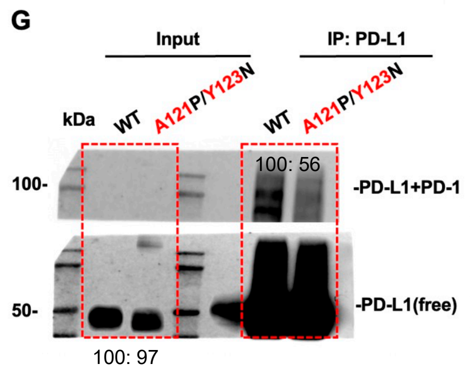


PD-L1 and PD-1 expression in HMVECs and TALL-104 cells

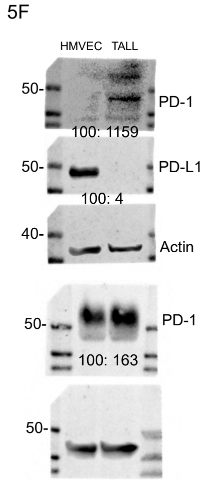


Co-IP and WB analysis of the interaction between PD-L1 (HMVECs) and PD-1 (TALL-104 cells) after co-culture in the presence of YKL-40

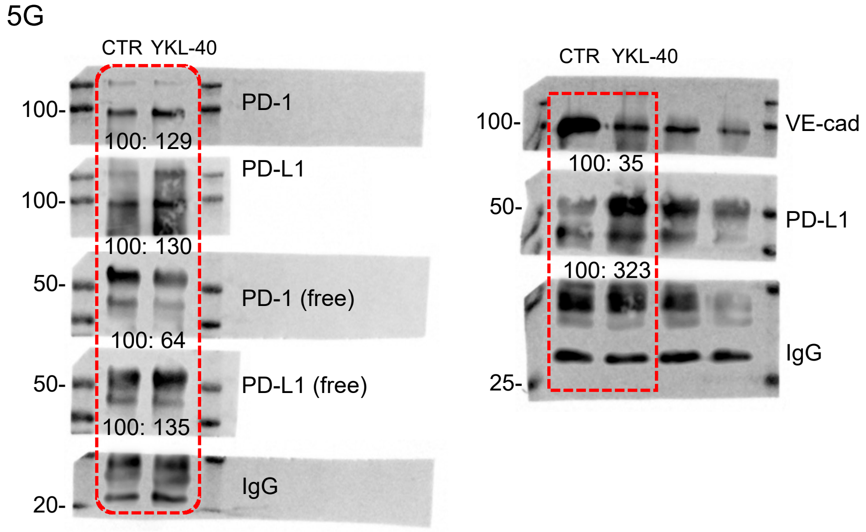


Cellular PD-L1 and VE-cad expression were analyzed by immunoblotting

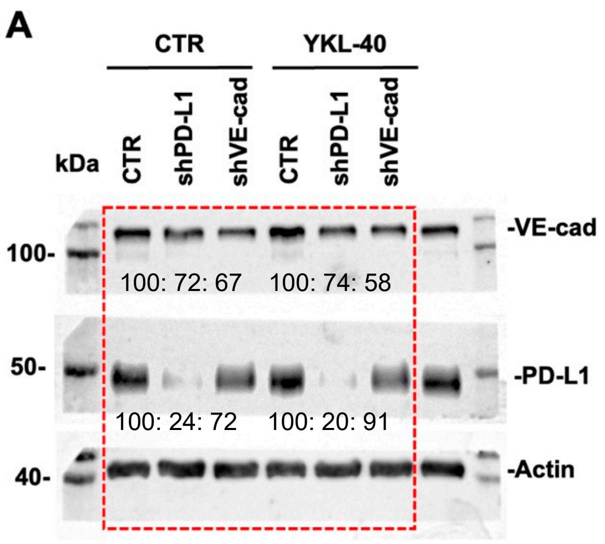


CCR5 protein levels in HMVECs

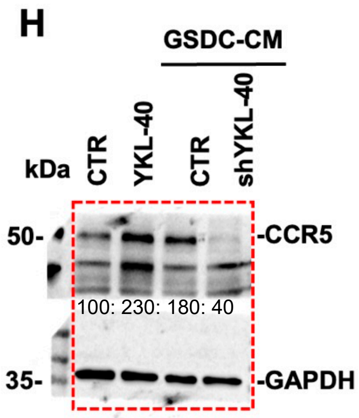


The interaction of CCR5 with VE-cad in HMVECs treated with YKL-40 (200 ng/mL) was analyzed by Co-IP and WB

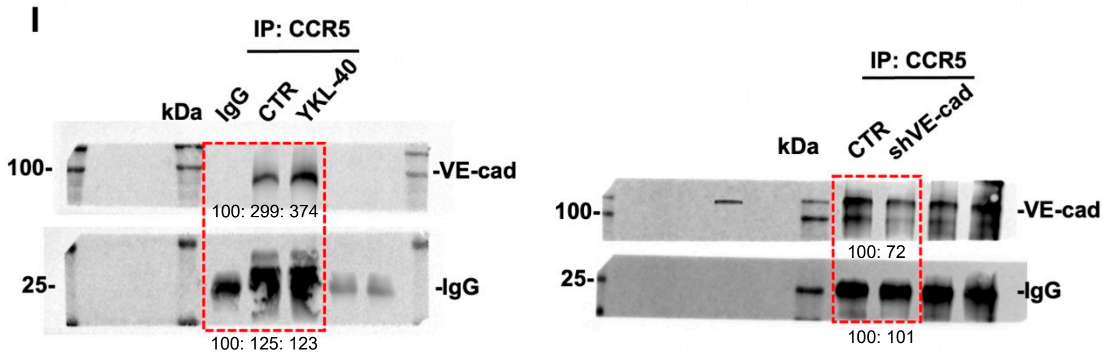


HMVECs were incubated with YKL-40 (200 ng/mL), TAK-652 (30 nM) or combination for 8 hours. Akt and MAPK pathway were analyzed by immunoblotting.

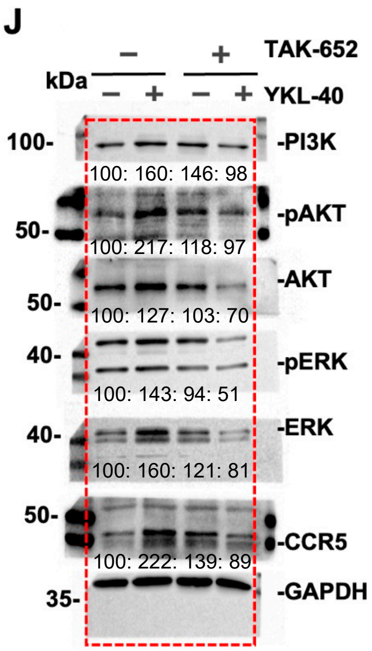


HMVECs were incubated with condition medium (CM) from GSDCs that were stably transfected with shYKL-40 or CTR shRNA. PD-L1 and VE-cad protein expression were visualized via Western blotting (WB)

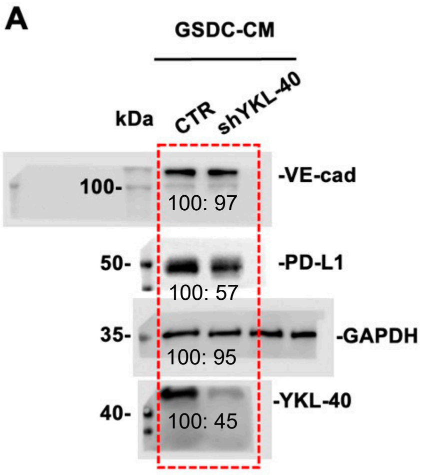


Lysates from GSDCs and GL261 cells were collected to evaluate expression of YKL-40, Vim and SMa. Immunocytochemistry of Vim in both cell lines was shown

B

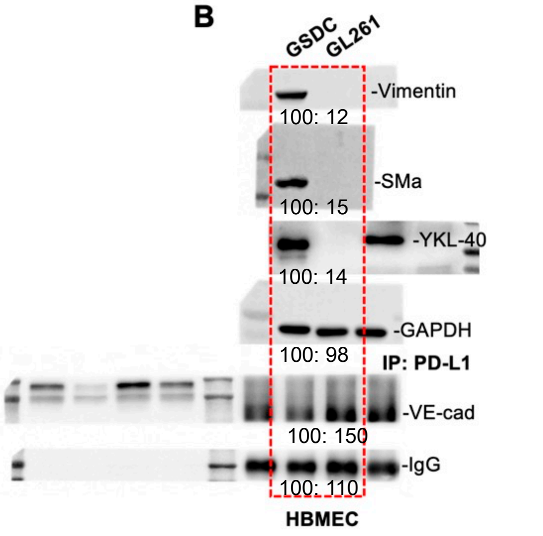


293 T cells were treated overnight with YKL-40 (200 ng/mL) and cell lysates were then subjected to Co-IP and WB.

C

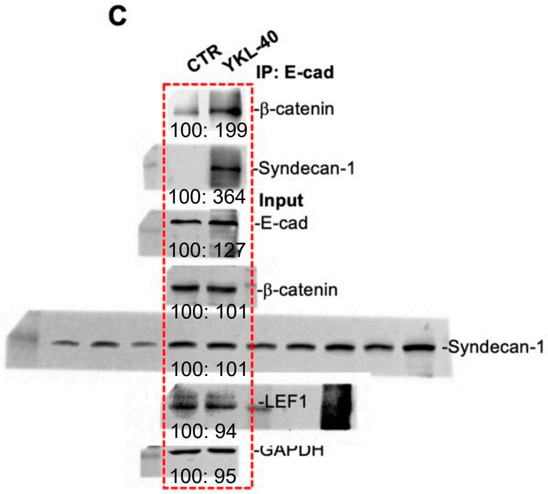


Three shRNA PD-L1 and two shRNA VE-cad cell lines were subjected to evaluation of PD-L1 and VE-cad expression via WB, in which shPD-L1 (#3) and shVE-cad (#1) cell lines were selected for further study.

B

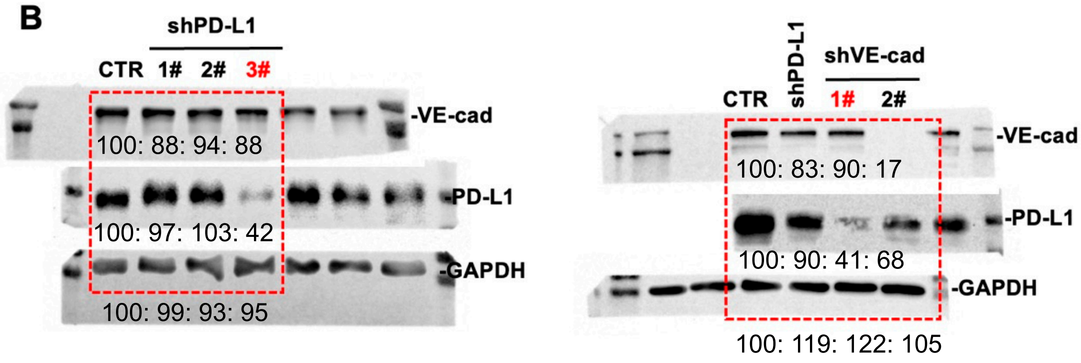

Co-IP and WB analysis of the interaction between PD-L1 and VE-cad in shRNA HMVECs in the presence of YKL-40.

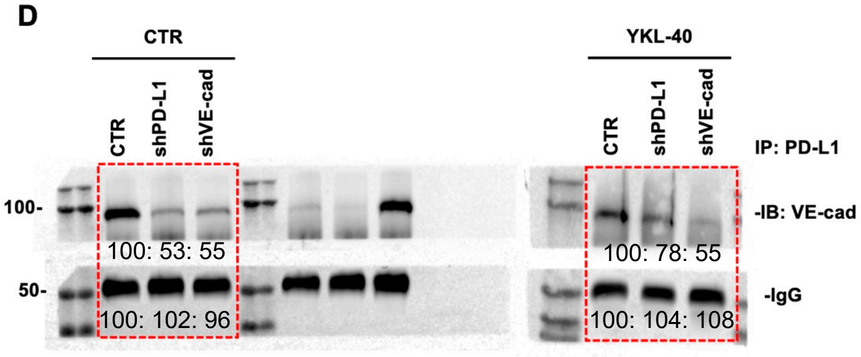

Supplement: Supplementary file 1 [file cancers-15-04257-s001.zip › File S1 for Western blot.pdf]
