# Supplementary material for: Vascular Immune Evasion of Mesenchymal Glioblastoma Is Mediated by Interaction and Regulation of VE-Cadherin on PD-L1
Source: Cancers (Basel). 2023 Aug 25;15(17):4257. doi: 10.3390/cancers15174257 (PMC10486786; doi:10.3390/cancers15174257)
Supplement: Supplementary file 1 [file cancers-15-04257-s001.zip › supplementary figures.pdf]

Supplementary Data for

Vascular Immune Evasion of Mesenchymal Glioblastoma Is Mediated by Interaction and Regulation of VE-Cadherin on PD-L1

Jing Luo *et al.*

Figs. S1 to S5:

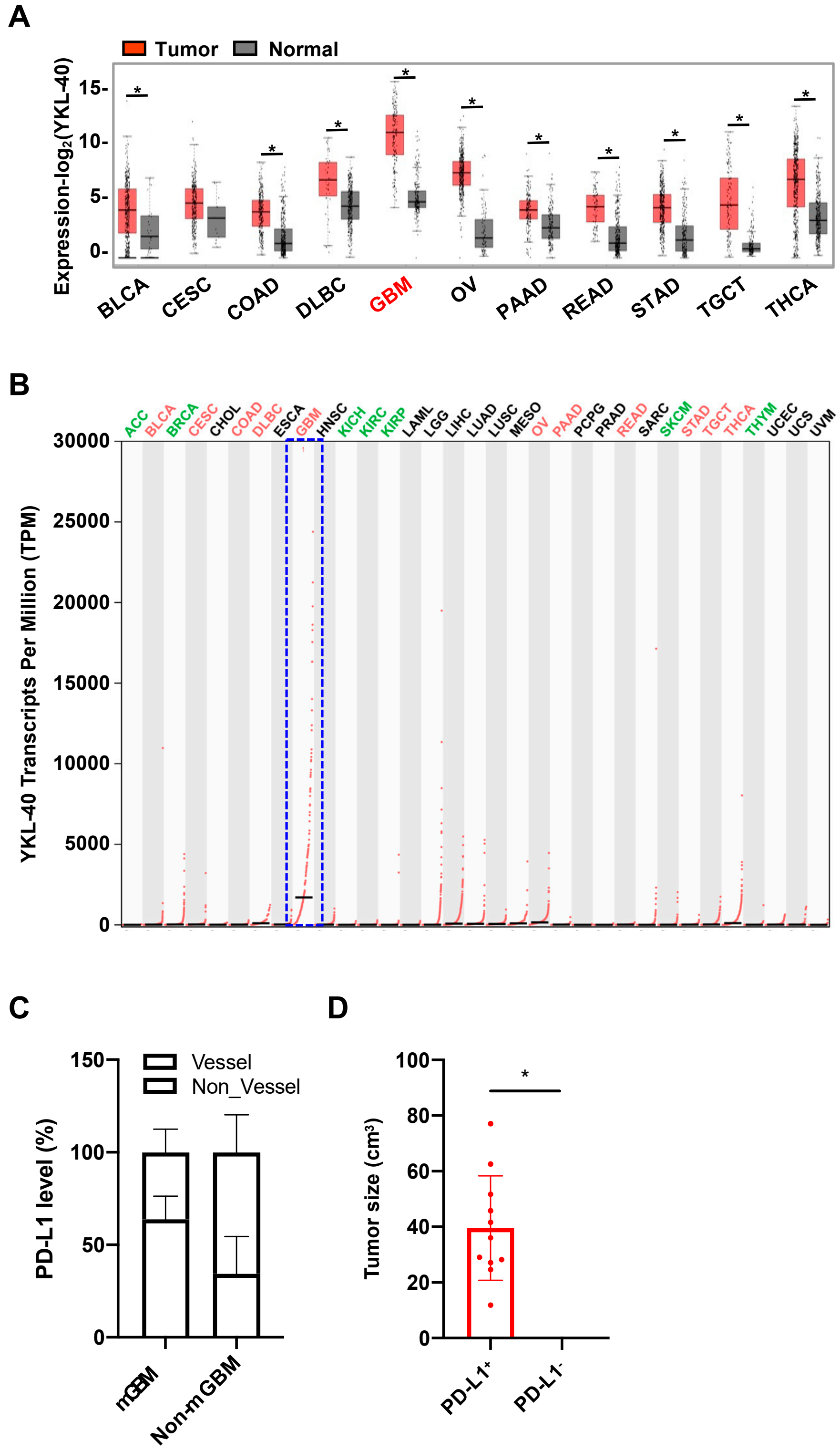

Figure S1. Expression of YKL-40 and PD-L1 expression in human cancers. (**A**) Database analysis (http://gepia.cancer-pku.cn) of YKL-40 levels in multiple human cancers, including bladder urothelial carcinoma, BLCA (Normal vs. Tumor, 28 vs. 404), cervical squamous cell carcinoma and endocervical adenocarcinoma, CESC (13 vs. 306), colon adenocarcinoma, COAD (349 vs. 275), lymphoid neoplasm diffuse large B-cell lymphoma, DLBC (337 vs. 47), glioblastoma, GBM (207 vs. 163), ovarian serous cystadenocarcinoma, OV (88 vs. 426), pancreatic adenocarcinoma, PAAD (171 vs. 179), rectum adenocarcinoma, READ (318 vs. 92), stomach adenocarcinoma, STAD (211 vs. 408), testicular germ cell tumors, TGCT (165 vs. 137), thyroid carcinoma, THCA (337 vs. 512), *$p<0.05$. (**B**) YKL-40 transcript levels were the highest in GBM relative to 33 human cancers. **(C)** Vascular PD-L1 levels in mGBM (n=6) and non-mGBM tumors (n=4). Data were presented as mean with SEM. Statistical significance was analyzed by *t*-test, *$p<0.05$. (**D**) PD-L1 expression was correlated with tumor

size. PD-L1-positive (sore ≥3, n=10) vs. PD-L1-negative GBM (sore<3, n=28). Tumor size was calculated: Volume = Length × Width² (or Width × Height) × 0.52 (cm³).

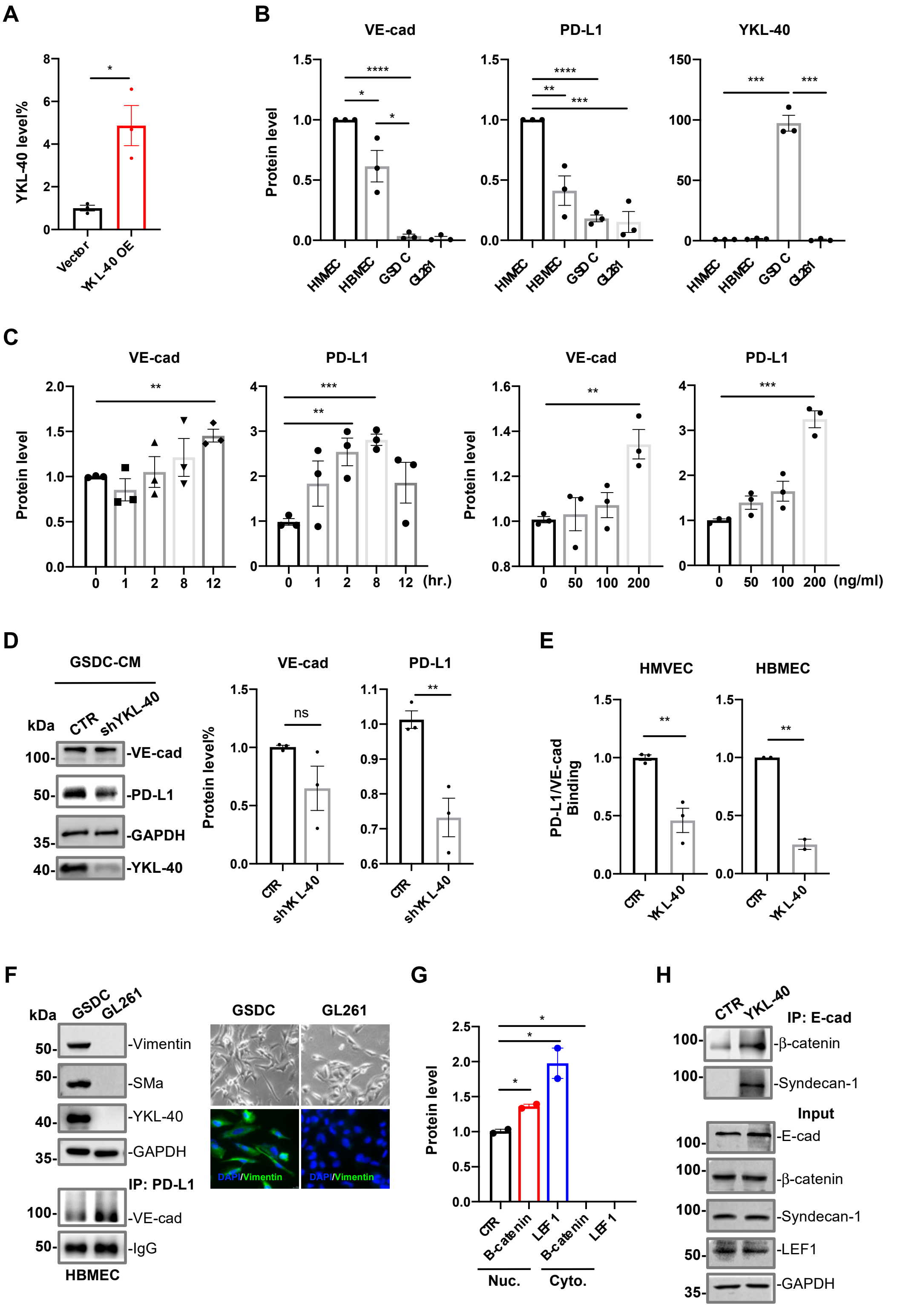

Figure S2. YKL-40 upregulates PD-L1 through activation of the VE-cad/β-catenin/LEF pathway in endothelial cells. (A) Lysates from GL261 control and YKL-40 OE cells were collected to evaluate expression of YKL-40 (mean ± SEM, n=3). (**B**) Quantification of PD-L1, VE-cad and YKL-40 expression in endothelial cell lines (HMVECs and HBMECs) and glioma cell lines (GSDC and GL261) by western blot assay shown in Fig. 3A (mean ± SEM, n=3). (**C**) Quantification of PD-L1 and VE-card level in HMCECs incubated with YKL-40 at a time course and different doses shown in Fig. 3C (mean ± SEM, n=3). (**D**) HMVECs were incubated with condition medium (CM) of GSDCs which were transfected with shYKL-40 or CTR shRNA. PD-L1 and VE-cad expression were determined

(mean ± SEM, n=3). (**E**) Quantification of binding between PD-L1 and VE-cad in HMVECs and HBMECs shown in Fig. 3E. (**F**) Lysates from GSDCs and GL261 cells were collected to evaluate expression of YKL-40, Vim and SMa. Immunocytochemistry of Vim in both cell lines was shown. Scale bars, 50 μm. Conditioned media (CM) was collected from each cell population and applied to HBMECs followed by Co-IP and WB of VE-cad and PD-L1 to evaluate protein-protein interaction. (**G**) Quantification of β-catenin and LEF1 level in nucleus and cytoplasm of HMVECs incubated with YKL-40 shown in Fig. 3H (mean ± SEM, n=2). (**H**) 293 T cells were treated overnight with YKL-40 (200 ng/mL) and cell lysates were then subjected to Co-IP and WB. Interaction of E-cad with β-catenin or syndecan-1 by Co-IP and WB (top), and expression of E-cad, β-catenin, Syndecan-1, LEF1 and GAPDH by WB (bottom). The uncropped blots are shown in File S1.

Statistical significance was analyzed by *t*-test; *p<0.05, **p<0.01, ***p<0.001, ****p<0.0001.

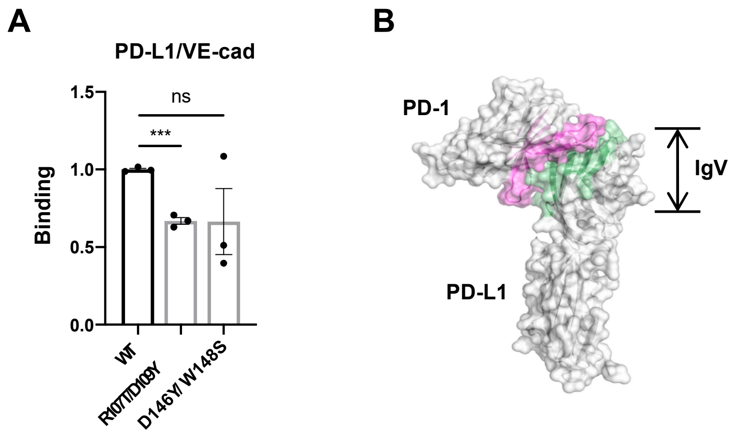

Figure S3. VE-cad and PD-1 bind to the same region of PD-L1. (A) Quantification of binding. between PD-L1 and VE-cad or mutants shown in Fig. 4E (mean ± SEM, n=3). ***p<0.001. **(B)** Surface representation of the hPD-1/hPD-L1 complex structure (PDB: 4ZQK). The interaction regions of hPD-1 and hPD-L1 (IgV domain) were represented by violet and green, respectively. Statistical significance was analyzed by *t*-test; ***p<0.001.

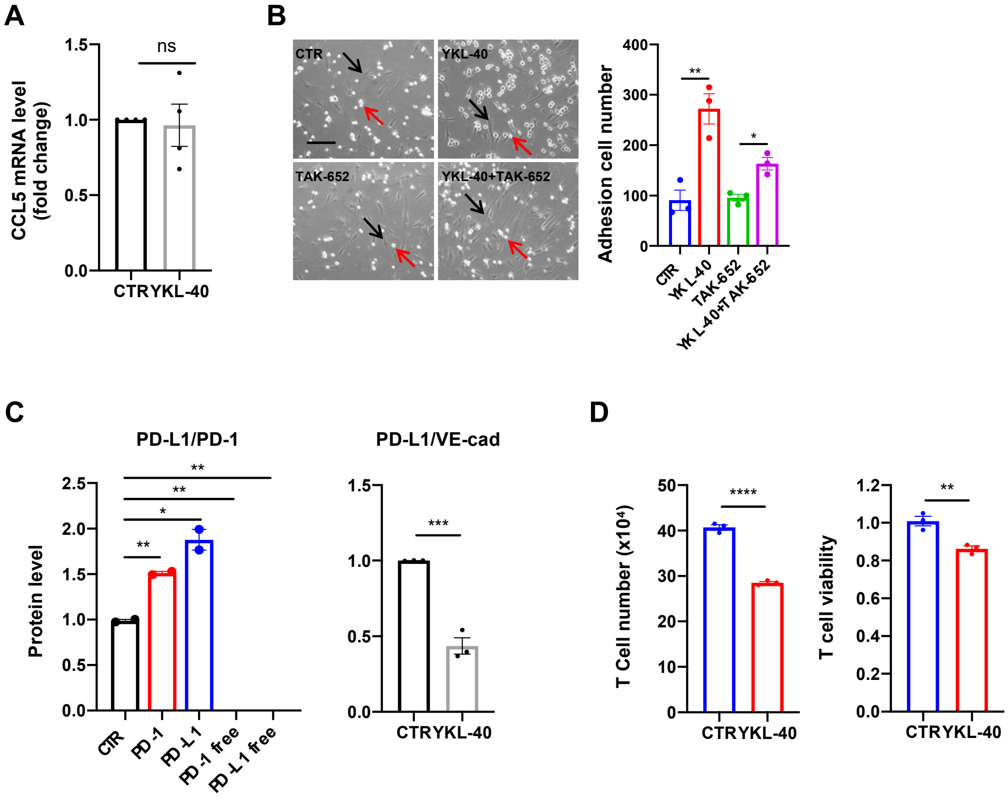

Figure S4. Cell adhesion and the interaction between endothelial cells and TALL-104 cells are mediated by interaction of PD-1/PD-L1. (A) The mRNA level of C-C chemokine ligand 5 (CCL5) in TALL-104 cells treated with or without YKL-40 (200 ng/mL) (mean ± SEM, n=3). (B) HMVECs co-cultured with TALL-104 cells in the indicated conditions (YKL-40: 200 ng/mL, TAK-652: 30 nM). The representative images of cell adhesion (left) and qualifications of the adhesion TALL-104 cells (right). Scale bars, 50 μm. Black arrows indicated HMVECs and red arrows showed TALL-104 cells (mean ± SEM, n=3). (C) Quantification analysis of the interaction between PD-L1 (HMVECs) and PD-1 (TALL-104 cells) after co-culture in the presence of YKL-40 and cross-link using 4% para-formaldehyde (left) shown in Fig. 5G left; Quantification analysis of the interaction between PD-L1 and VE-cad in HMVECs after co-cultured with TALL-104 (right) shown in Fig. 5G right (mean ± SEM, n=3). (D) Cell number and viability of TALL-104 after co-culture with HMVECs in the presence of YKL-40 via cell number counting and CCK-8 assay, respectively (mean ± SEM, n=3). Statistical significance was analyzed by *t*-test; **p*<0.05, ***p*<0.01, ****p*<0.001, *****p*<0.0001.

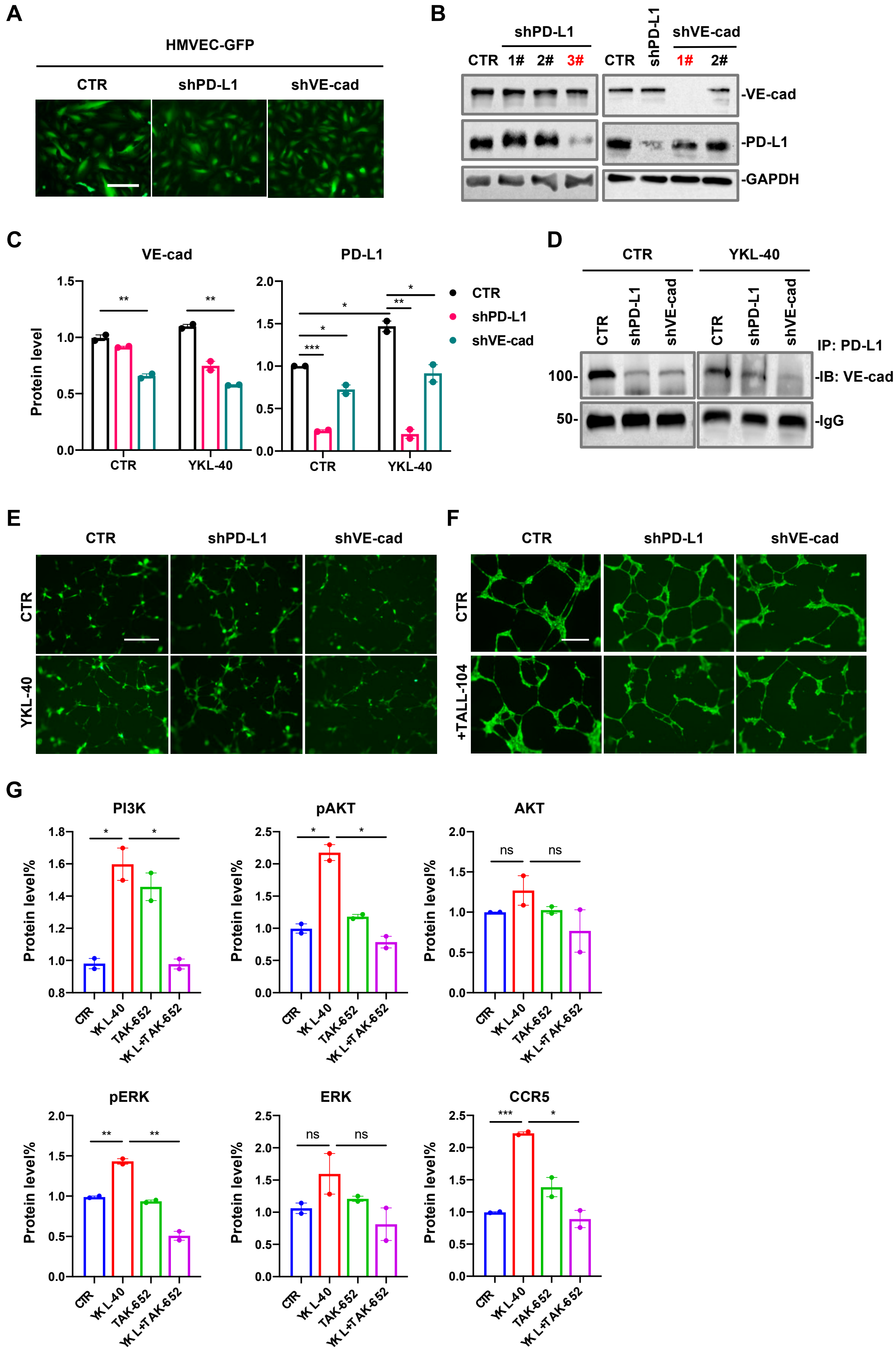

Figure S5. shPD-L1 and shVE-cad in HMVECs restores the cytotoxic activity of CTLs. (A) IF images of GFP-positive shPD-L1 and shVE-cad HMVEC cell lines. Scale bars, 50 μm. **(B)** Three shRNA PD-L1 and two shRNA VE-cad cell lines were subjected to evaluation of PD-L1 and VE-cad expression via WB, in which shPD-L1 (#3) and shVE-cad (#1) cell lines were selected for further study. (**C**) Quantification of cellular PD-L1 and VE-cad expression after HMVECs stably expressing shPD-L1, shVE-cad or CTR were treated with YKL-40 (200 ng/mL) for 8 hours shown (in Fig. 5A) (mean ± SEM, n=2). (**D**) Co-IP and WB analysis of the interaction between PD-L1 and VE-cad in shRNA HMVECs in the presence of YKL-40. **(E)** Analysis of tubes formed by YKL-40-treated shPD-L1, shVE-cad or CTR HMVECs expressing GFP. Scale bars, 100 μm. **(F)** shPD-L1, shVE-cad or CTR

HMVECs were co-cultured with TALL-104 cells, and then were analyzed for the tube formation. GFP images of HMVECs. Scale bars, 50 μm. **(G)** Quantification analysis of Fig. 6J (mean ± SEM, n=2). Statistical significance was analyzed by *t* test; *$p<0.05$, **$p<0.01$, ***$p<0.001$. The uncropped blots are shown in File S1.
